# Transformer-based fusion of radiomics-habitat and deep learning for assessing unruptured intracranial aneurysm instability

**DOI:** 10.3389/fnins.2026.1818110

**Published:** 2026-06-17

**Authors:** Linghao Li, Shanshan Xie, Xinmei Ma, Wentao Gong, Yuncai Ran, Xiao Wang, Jinyi Li, Jing Li, Yong Zhang, Sheng Guan, Qichang Fu

**Affiliations:** 1 Department of Magnetic Resonance, The First Affiliated Hospital of Zhengzhou University, Zhengzhou, China; 2 Department of Interventional Neuroradiology, The First Affiliated Hospital of Zhengzhou University, Zhengzhou, China

**Keywords:** aneurysm instability, multi-modal fusion, radiomics-habitat, transformer, unruptured intracranial aneurysms

## Abstract

**Objectives:**

To develop and validate a prediction model that integrates radiomics-habitat and deep learning (DL) features derived from vessel wall MRI (VWI) for evaluating unruptured intracranial aneurysms (UIAs) instability.

**Methods:**

First, from January 2022 to January 2024, 519 consecutive patients with suspected UIAs were screened. After applying exclusion criteria, 293 patients with 312 UIAs were ultimately enrolled. 197 UIAs were stable (from 188 patients) and 115 UIAs were unstable (from 105 patients). Second, aneurysm regions were segmented, and K-means clustering was used to partition them into three habitat subregions. Third, a Transformer-based fusion model for assessing UIA instability was developed to integrate radiomics-habitat features, DL features, and clinical variables. Model performance was evaluated using AUC, calibration curves, and clinical gain metrics, including Net Reclassification Index (NRI) and Integrated Discrimination Improvement (IDI). Last, SHAP (SHapley Additive exPlanations) was applied to enhance model interpretability.

**Results:**

The Transformer-based fusion model assessing UIA instability exhibited superior performance (validation AUC = 0.844) compared with the optimal radiomics-habitat model (AUC = 0.721) and the top-performing DL model (DenseNet169, AUC = 0.816). The model demonstrated superior clinical utility, with an NRI of 0.282 and an IDI of 0.558 compared to the Radiomics-Habitat model. Decision curve analysis showed a high net clinical benefit across a range of threshold probabilities.

**Conclusion:**

The Transformer-based fusion model provides an exploratory risk-assessment model and has the potential to assist in clinical decision-making.

## 1 Introduction

Subarachnoid hemorrhage (SAH) resulting from intracranial aneurysm (IA) rupture is a leading cause of severe cerebrovascular events and carries substantial mortality. Its abrupt onset and poor prognosis remain a persistent burden to public health (Thompson et al., 2015; Laukka et al., 2024; Long et al., 2025). Precise pre-rupture evaluation of IA stability and early identification of high-risk lesions are critical steps in optimizing personalized clinical intervention strategies and improving patient outcomes, which constitutes an unmet clinical need in the management of unruptured intracranial aneurysms (UIAs) (Society of Neurosurgery of Chinese Medical Association, 2024).

Current evaluation of UIAs stability relies on aneurysm size, morphological parameters, and select clinical risk factors. Although digital subtraction angiography (DSA) provides high-resolution visualization of intravascular anatomy, it lacks the capacity to reveal critical details of aneurysm wall tissue composition or inflammatory responses. Vessel wall MRI (VWI) delivers superior spatial resolution and excellent soft-tissue contrast. It can clearly delineate aneurysm wall architecture and enhancement characteristics, thereby furnishing a more comprehensive imaging foundation for stability assessment (Samaniego et al., 2019; Santarosa et al., 2020; Zhou et al., 2025; Benndorf et al., 2023).

Radiomics provides high-throughput extraction of deep phenotypic data by treating a Region of Interest (ROI) as a holistic entity. Using geometric and texture-based features, it characterizes macroscopic heterogeneity for risk stratification (Sohrabi-Ashlaghi et al., 2024; Yan et al., 2025; Liu et al., 2021; Dier et al., 2025). However, averaging features across the entire lesion often masks subtle local variations. To address this, habitat imaging utilizes unsupervised clustering—such as K-means—to partition tumors into distinct subregions. By grouping voxels with similar imaging signatures, this approach identifies microenvironmental variations that are otherwise obscured in whole-lesion analysis (Gatenby et al., 2013).

Deep learning provides powerful alternatives for medical image analysis. Convolutional Neural Networks (CNNs) utilize local receptive fields and spatial hierarchies to automatically capture fine-grained textures and anatomical morphologies. Recently, multimodal and multi-omics fusion frameworks have been developed for UIA risk stratification, among which a representative study adopted integrated multi-feature fusion modeling for aneurysm risk prediction (Turhon et al., 2023). However, most existing methods, including the above multimodal fusion strategy, merely perform simple feature concatenation without modeling intralesional spatial heterogeneity of the aneurysm wall and lack advanced attention mechanisms to capture high-order interactions among heterogeneous features, thus failing to fully mine the biological information contained in VWI. In clinical practice, radiologists can only subjectively assess obvious morphological abnormalities, while AI technology can noninvasively quantify subtle wall heterogeneity and inflammatory phenotypes invisible to the naked eye, rather than merely reducing the workload of radiologists. In contrast, the Transformer architecture serves as an advanced platform for heterogeneous feature integration. By projecting data into a unified semantic space and employing multi-head attention, Transformers model global dependencies and complex non-linear interactions across modalities, which holds great potential to overcome the limitations of conventional fusion strategies (Zhang et al., 2025).

We hypothesize that fusing clinical variables, radiomics-habitat features, and deep learning (DL) features via a Transformer framework can holistically capture the clinical correlates, spatial heterogeneity, and high-dimensional imaging phenotypes of IAs, thereby enabling more accurate UIAs stability evaluation. To test this hypothesis, we developed and compared three models (a radiomics-habitat model, a DL model, and a Transformer fusion model) using VWI data, aiming to systematically evaluate the utility of multi-modal features for UIAs instability prediction—ultimately seeking to deliver a non-invasive, robust risk stratification tool to support clinical decision-making.

## 2 Materials and methods

### 2.1 Ethics statement

This study was approved by the Ethics Committee of The First Affiliated Hospital of Zhengzhou University (Ethics No.: SS-2018-11). Given the retrospective nature of this study, the requirement for informed consent was exempted. All procedures strictly adhered to the principles of the Declaration of Helsinki. Patient privacy and data security were ensured through data encryption and restricted access.

### 2.2 Study population

We retrospectively included patients diagnosed with IAs who underwent high-resolution SPACE sequence scanning at a tertiary Grade-A medical institution between January 2022 and January 2024.

### 2.3 Inclusion and exclusion criteria

Determination was jointly completed by two senior neuroradiologists (with 10 and 8 years of experience in neurointerventional radiology) and one neurointerventionist (12 years of experience), all blinded to the model training process. Disagreements were resolved via a third senior neuroradiologist (15 years of experience) who was not involved in the initial assessment.

*Inclusion Criteria*: (1) Availability of comprehensive follow-up imaging (including DSA or VWI); (2) Successful completion of plain and contrast-enhanced VWI, with image quality meeting the requirements for subsequent feature extraction and analysis; (3) Complete clinical data (including demographics, history of underlying diseases, medication history, etc.) and imaging data; (4) No severe underlying diseases affecting imaging, such as severe hepatic or renal dysfunction or coagulation disorders. Patients meeting any of the following were included in the unstable group: (1) Sudden and severe headache on the ipsilateral side of the aneurysm within 2 weeks prior to admission, with no history of headache in the past 5 years; (2) Sudden headache on the ipsilateral side of the aneurysm within 1 month prior to admission, accompanied by one or more symptoms of pupil light reflex disappearance, ptosis, or extraocular muscle paralysis; (3) Previous examination within 12 months indicating an increase in aneurysm diameter or appearance of a daughter sac; (4) Aneurysm rupture occurring within 3 months after the examination, or aneurysm progression found in two consecutive examinations within 6 months (Edjlali et al., 2014).

**Figure 1:**
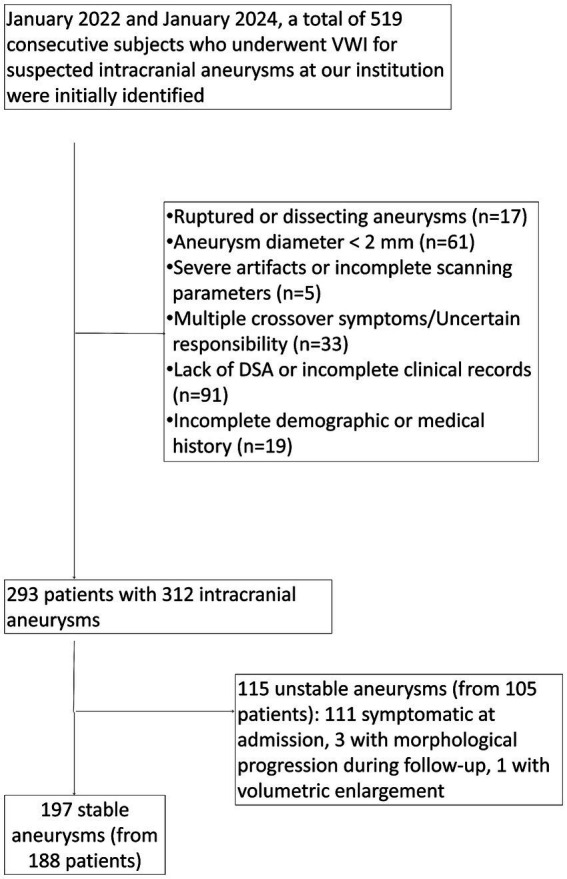
Flowchart of the study population recruitment. From January 2022 to January 2024, 519 consecutive patients with suspected IAs were screened. After applying the exclusion criteria, 293 patients with 312 aneurysms were finally enrolled in the study.

*Exclusion Criteria*: (1) Presence of ruptured or dissecting aneurysms; (2) Aneurysm diameter less than 2 mm; (3) Severe artifacts, motion blur, or incomplete scanning parameters preventing Region of Interest (ROI) delineation; (4) Subjects with aneurysms presenting multiple crossover symptoms (uncertainty regarding which aneurysm was responsible for specific symptoms); (5) Lack of DSA imaging or incomplete clinical records; (6) Subjects with incomplete demographic and medical history data. The inclusion and exclusion process is detailed in Figure 1.

### 2.4 Image acquisition and preprocessing

1. Image Acquisition All patients were scanned using 3.0 T MRI equipment (Siemens MAGNETOM Prisma, Siemens MAGNETOM Vida). Scanning sequences included high-resolution SPACE T1-weighted sequences (pre- and post-contrast) and 3D-TOF MRA sequences. Specific parameters are shown in Supplementary materials.
2. Image Preprocessing Image data were standardized and preprocessed using PyRadiomics software (version 3.3.0) and Python (version 3.9.0): (1) DICOM images were converted to NIfTI format for batch analysis; (2) *Z*-score normalization was applied to correct grayscale values and eliminate differences caused by different equipment and scanning parameters; (3) Gaussian filtering (sigma = 1.0) was used to reduce image noise, and morphological operations were applied to eliminate motion and pulsation artifacts; (4) N4 bias field correction was performed to eliminate grayscale inhomogeneity, and rigid registration was used for spatial alignment; (5) Images were resampled to an isotropic voxel size of 0.5 × 0.5 × 0.5 mm^3^ using the sitkBSpline interpolation function; (6) Pre- and post-contrast images were used as dual-channel input data and uniformly cropped to a standardized size of 96 × 96 × 96 voxels for deep learning model training.

### 2.5 ROI delineation and habitat generation

#### 2.5.1 ROI delineation

Two physicians with over 5 years of experience in neuroimaging diagnosis manually delineated ROIs slice-by-slice using ITK-snap software (version 3.8.0) based on pre- and post-contrast SPACE T1 sequences, under blinded conditions (unaware of patient clinical information and outcomes). The delineation scope included the aneurysm wall, intratumoral thrombus (if present), and the proximal segment of the parent artery [(1). For aneurysms with a diameter smaller than the length of the responsible vessel segment, the corresponding vessel segment was delineated; (2). For aneurysms with a diameter larger than the length of the responsible vessel segment, a length of the responsible vessel equal to the aneurysm length was delineated], strictly excluding surrounding normal brain tissue and cerebrospinal fluid. To ensure consistency, the two physicians first jointly delineated ROIs for 10 patients to unify standards before independently completing the remaining cases. One month later, images of 30 randomly selected patients were re-delineated by the same physician to assess intra-observer consistency. Consistency was quantified using the Dice Similarity Coefficient (DSC) and Intraclass Correlation Coefficient (ICC), with DSC ≥ 0.85 and ICC ≥ 0.8 considered good consistency.

#### 2.5.2 Habitat generation

For the preprocessed post-contrast ROI images, first-order features and texture features (including GLCM, GLRLM, GLSZM, GLDM, NGTDM) were extracted for each voxel. K-means clustering algorithm was used to perform cluster analysis on the ROI voxels of pre- and post-contrast sequences separately. The number of clusters was set to 2–9, and the optimal number was determined via the Calinski-Harabasz index, Davies-Bouldin index, and Silhouette Coefficient. The optimal number of clusters was determined to be 3. Calinski-Harabasz index, Davies-Bouldin index, and Silhouette Coefficient for each clusters can be found in Supplementary Table 1.

### 2.6 Feature extraction and screening

#### 2.6.1 Features extraction

- Clinical Features: A total of 12 clinical and imaging indicators were extracted, including age, gender, aneurysm size, location, history of hypertension, diabetes, hyperlipidemia, smoking, alcohol consumption, aspirin use, history of SAH, and AWEP score. AWEP (Aneurysm Wall Enhancement Pattern) was defined as: 0—no wall enhancement; 1—focal wall enhancement; 2—circumferential wall enhancement. Aneurysm location was quantified using the PHase score: 0—Internal Carotid Artery; 2—Middle Cerebral Artery; 4—Anterior Cerebral Artery, Posterior Cerebral Artery, Anterior/Posterior Communicating Artery.
- Radiomics-Habitat Features: Traditional radiomics features were extracted from the global ROI containing the aneurysm and parent artery (pre- and post-contrast); habitat features were extracted from the 3 habitat subregions separately. Traditional radiomics features and habitat features were directly mixed to form the radiomics-habitat feature set.
- Deep Learning Features: Dual-channel images were input into pre-trained deep learning models (DenseNet169, ResNet50, ResNet101, DenseNet121, ShuffleNet). Features from the layer preceding the fully connected layer of each model were extracted and compressed into a deep learning feature set using Principal Component Analysis (PCA). Extract the 1,024-dimensional features of the penultimate layer of DenseNet169, reduce the PCA to 64 dimensions. The proportion of explained variance is ≥95%. More details can be found in Supplementary material.

#### 2.6.2 Feature screening

ICCs were calculated for features in the training set, retaining those with ICC ≥ 0.80. Pearson correlation analysis was performed, and for feature pairs with a correlation coefficient |*r*| ≥ 0.90, the one with the larger variance was retained to eliminate multicollinearity. For the radiomics-habitat dataset, LASSO regression (10-fold cross-validation) was used to screen the remaining features, setting the penalty coefficient *λ* to retain features with non-zero regression coefficients, forming the optimal feature set.

### 2.7 Model construction

#### 2.7.1 Radiomics-Habitat model construction

Using the screened radiomics-habitat feature set as input, five machine learning models were constructed: Logistic Regression (LR), Support Vector Machine (SVM), Random Forest (RF), XGBoost, and LightGBM. Model parameters were optimized via grid search with 5-fold cross-validation to identify the optimal algorithm and hyperparameter configuration for performance maximization.

#### 2.7.2 Deep learning model construction

Deep learning models for extracting, including DenseNet121, DenseNet169, ResNet50, ResNet101, and ShuffleNet, were developed based on dual-channel images (integrating pre- and post-contrast phases). The input layer was configured to 96 × 96 × 96 × 2 (H × W × D × C). The architecture comprised corresponding convolutional, pooling, and fully connected layers, culminating in an output layer with a sigmoid activation function to estimate the probability of aneurysm instability.

#### 2.7.3 Transformer-based fusion model

**Figure 2:**
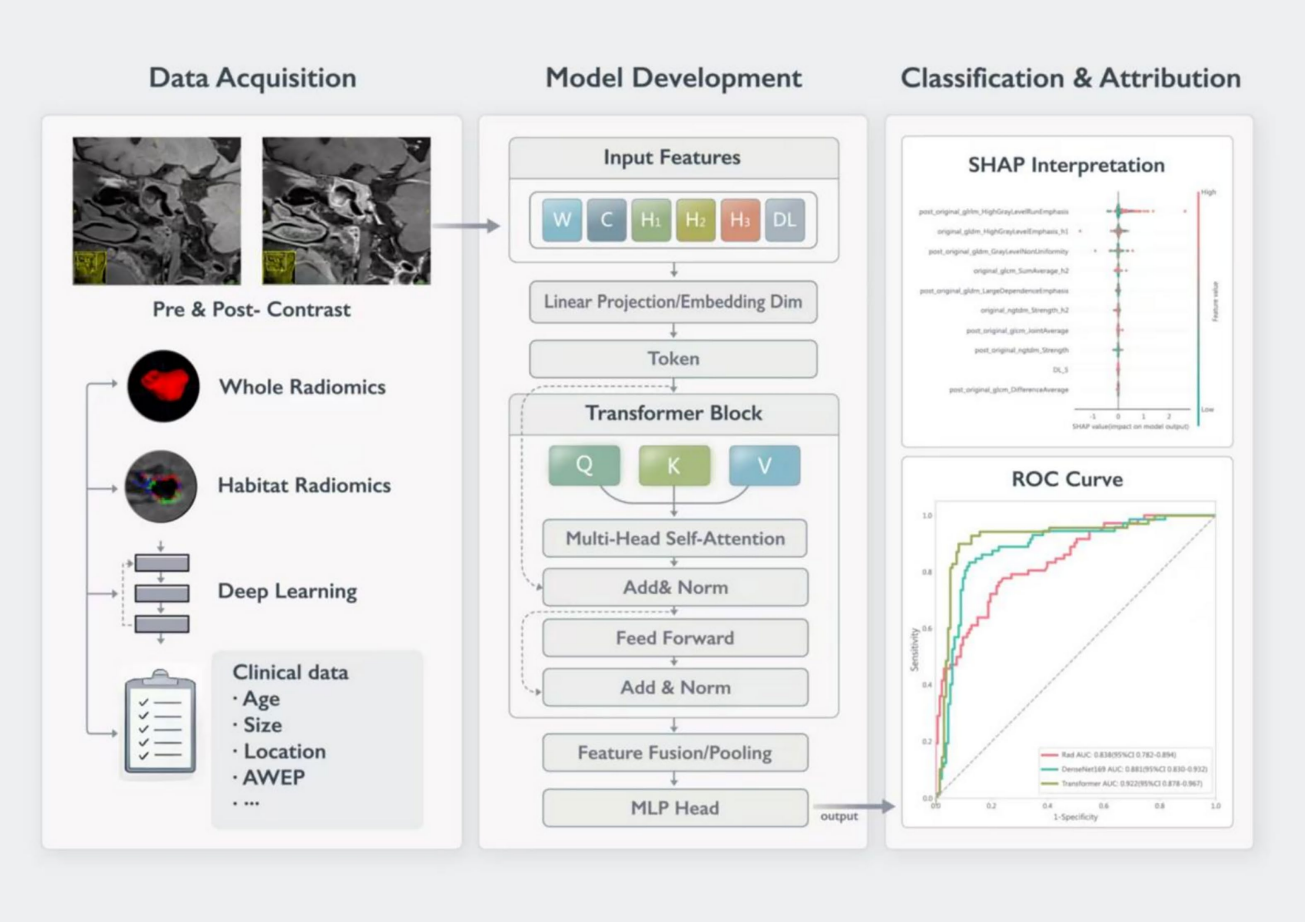
Schematic framework of the proposed multimodal Transformer pipeline, comprising three phases: (1) Data acquisition: Multi-dimensional features (whole-radiomics, habitat radiomics, deep learning features) were extracted from pre- and post-contrast MRI scans, with integration of clinical characteristics (age, size, location, AWEP). (2) Model development: A Transformer-based architecture was designed for heterogeneous feature fusion; diverse inputs (W: whole radiomics; C: clinical data; H1–H3: habitat features; DL: deep learning features) were embedded as tokens, processed via multi-head self-attention modules and feed-forward networks to capture inter-feature dependencies, and an MLP head was adopted for final output. (3) Classification and attribution: Model performance was evaluated using ROC curves and other metrics; SHAP analysis was applied for model interpretation and visualizing the contribution of top features. AWEP, aneurysm wall enhancement pattern; MLP, multilayer perceptron; ROC, receiver operating characteristic; SHAP, SHapley Additive exPlanations.

A Transformer-based fusion model was constructed to integrate clinical, radiomics-habitat, and deep learning features. The model architecture and training parameters were show in Supplementary material. The integrated framework for feature extraction and model development is summarized in Figure 2.

### 2.8 Statistical analysis

Statistical analyses were conducted using Python (version 3.9.0). Quantitative data were first assessed for normality via the Shapiro–Wilk test; those conforming to a normal distribution were compared between groups using the independent samples *t*-test, while non-normally distributed data were analyzed using the Wilcoxon rank-sum test (Mann–Whitney *U* test). Categorical variables were presented as counts (percentages) and compared using Pearson’s *χ*^2^ test.

Model performance was evaluated using the following validated metrics: accuracy, area under the receiver operating characteristic curve (AUC), sensitivity, specificity, positive predictive value (PPV), negative predictive value (NPV), and F1 score (harmonic mean of precision and recall). Pairwise differences in AUC values between models were statistically compared using the DeLong test. Calibration curves were generated to assess the agreement between model-predicted probabilities and observed clinical outcomes. Decision curve analysis (DCA) was performed to evaluate clinical utility by calculating the net benefit across a predefined range of risk thresholds (0.1–0.9), a range clinically relevant for aneurysm intervention decisions.

Net Reclassification Improvement (NRI) and Integrated Discrimination Improvement (IDI) were computed to quantify the incremental value of the Transformer fusion model: NRI assessed improvements in classification decision-making, while IDI measured enhancements in the statistical discriminatory ability of predicted probabilities. SHapley Additive exPlanations (SHAP) analysis was conducted to identify top predictive features driving the Transformer fusion model’s outputs and to interpret the model’s decision-making rationale—critical for enhancing clinical trust and adoptability. A two-tailed *p*-value < 0.05 was considered statistically significant for all analyses.

## 3 Results

### 3.1 Patient baseline characteristics

This study included 293 patients with 312 unruptured intracranial aneurysms. Stability was classified at the aneurysm level: 197 aneurysms were stable (from 188 patients) and 115 aneurysms were unstable (from 105 patients). Patients were randomly divided into a training set (205 cases, 218 aneurysms) and a validation set (88 cases, 94 aneurysms) at a 7:3 ratio. In both cohorts, AWEP grade, history of hyperlipidemia, and aspirin use were significantly different between the stable and unstable groups (all *p* < 0.05). Aneurysm size showed a significant difference in the training set but not in the validation set. No significant differences were observed in other demographic and clinical variables, including age, gender, hypertension, diabetes, and smoking history. Detailed baseline characteristics of all variables are presented in Table 1. Non-significant clinical parameters and comparisons between the training and validation sets are provided in Supplementary material.

**Table 1:** Baseline characteristics of patients in training and validation sets.

Feature name	Training data			*p*	Validation data			*p*
	All (205)	Stable (133)	Unstable (72)		All (88)	Stable (55)	Unstable (33)	
Size	6.43 (3.80, 8.60)	6.03 (3.27, 7.91)	7.19 (4.21, 9.34)	0.045	6.65 (3.49, 8.13)	6.14 (3.26, 7.85)	6.79 (3.60, 8.76)	0.928
AWEP				<0.001				0.015
0	122 (59.51)	90 (67.67)	32 (44.44)		45 (51.14)	32 (58.18)	13 (39.39)	
1	69 (33.66)	39 (29.32)	30 (41.67)		36 (40.91)	22 (40.00)	14 (42.42)	
2	14 (6.83)	4 (3.01)	10 (13.89)		7 (7.95)	1 (1.82)	6 (18.18)	
Hyperlipidemia				0.012				0.044
0	151 (73.66)	106 (79.70)	45 (62.50)		63 (71.59)	44 (80.00)	19 (57.58)	
1	54 (26.34)	27 (20.30)	27 (37.50)		25 (28.41)	11 (20.00)	14 (42.42)	
Aspirin				<0.001				<0.001
0	64 (31.22)	28 (21.05)	36 (50.00)		25 (28.41)	5 (9.09)	20 (60.61)	
1	141 (68.78)	105 (78.95)	36 (50.00)		63 (71.59)	50 (90.91)	13 (39.39)	

Comparisons of demographic and clinical variables between stable and unstable aneurysm groups. AWEP, aneurysm wall enhancement pattern.

### 3.2 ROI delineation consistency

ROI delineation consistency between the 2 senior neuroradiologists and by the same physician at different time points was good. Inter-observer DSC was 0.86 ± 0.03, and ICC was 0.83 ± 0.04; intra-observer DSC was 0.90 ± 0.02, and ICC was 0.86 ± 0.03. All met analysis requirements (DSC ≥ 0.85, ICC ≥ 0.80), ensuring the reliability and accuracy of subsequent feature extraction.

### 3.3 Radiomics-Habitat model performance

After feature screening, 13 key features (including 5 habitat-related and 8 traditional radiomics features) were selected via LASSO regression with 10-fold cross-validation. The LASSO coefficients and category attribution of these selected features are visualized in Supplementary material. Among eight machine learning algorithms, the Random Forest (RF) model demonstrated the best overall performance and generalization, achieving an AUC of 0.838 in the training set and 0.721 in the validation set, significantly outperforming other classifiers (Table 2).

**Table 2:** Classification performance of Radiomics-Habitat models.

model_name	Accuracy	Accuracy 95%CI	AUC	AUC 95%CI	Sensitivity	Specificity	PPV	NPV	F1	Task
LR	0.746	0.6984–0.7947	0.764	0.6939–0.8345	0.625	0.812	0.643	0.8	0.634	train
LR	0.659	0.6016–0.7179	0.697	0.5845–0.8095	0.727	0.618	0.533	0.791	0.615	val
SVM	0.839	0.7995–0.8798	0.877	0.8238–0.9293	0.847	0.835	0.735	0.91	0.787	train
SVM	0.67	0.6127–0.7285	0.665	0.5464–0.7836	0.515	0.764	0.567	0.724	0.54	val
KNN	0.712	0.6618–0.7636	0.808	0.7521–0.8645	0.833	0.647	0.561	0.878	0.67	train
KNN	0.648	0.5894–0.7079	0.607	0.4838–0.7294	0.364	0.818	0.545	0.682	0.436	val
RandomForest	0.771	0.7256–0.8174	0.838	0.7815–0.8939	0.764	0.774	0.647	0.858	0.701	train
RandomForest	0.693	0.6378–0.7495	0.721	0.6131–0.8293	0.636	0.727	0.583	0.769	0.609	val
ExtraTrees	0.707	0.6564–0.7587	0.771	0.7065–0.8356	0.653	0.737	0.573	0.797	0.61	train
ExtraTrees	0.625	0.5657–0.6859	0.674	0.5612–0.7875	0.909	0.455	0.5	0.893	0.645	val
XGBoost	0.927	0.8986–0.9569	0.982	0.9689–0.9947	0.986	0.895	0.835	0.992	0.904	train
XGBoost	0.659	0.6015–0.7178	0.712	0.6037–0.8206	0.818	0.564	0.529	0.838	0.643	val
LightGBM	0.824	0.7835–0.8659	0.907	0.8658–0.9474	0.875	0.797	0.7	0.922	0.778	train
LightGBM	0.67	0.6126–0.7284	0.727	0.6221–0.8313	0.758	0.618	0.543	0.81	0.633	val
MLP	0.79	0.7465–0.8348	0.783	0.7147–0.8519	0.625	0.88	0.738	0.812	0.677	train
MLP	0.693	0.6374–0.7496	0.696	0.5810–0.8108	0.636	0.727	0.583	0.769	0.609	val

Performance metrics (accuracy, AUC, sensitivity, specificity, PPV, NPV, F1) for different machine learning classifiers (LR, SVM, KNN, RF, ExtraTrees, XGBoost, LightGBM, MLP) in training and validation sets. The Random Forest (RF) model was selected as the optimal radiomics-habitat model.

### 3.4 Deep learning model performance

Among five dual-channel deep learning architectures, DenseNet169 achieved the best performance with a validation AUC of 0.816 (Table 3, Supplementary Figure 2), outperforming ResNet and ShuffleNet variants. Consequently, 64-dimensional deep features were extracted from the pre-final layer of DenseNet169 using PCA for subsequent model fusion.

**Table 3:** Classification performance of deep learning models.

Model name	Accuracy	Accuracy 95%CI	AUC	AUC 95%CI	Sensitivity	Specificity	PPV	NPV	F1	Task
ShuffleNet	0.712	0.6614–0.7638	0.736	0.6656–0.8058	0.681	0.729	0.576	0.808	0.624	train
ShuffleNet	0.591	0.5296–0.6534	0.642	0.5266–0.7577	0.879	0.418	0.475	0.852	0.617	val
resnet50	0.824	0.7837–0.8659	0.827	0.7653–0.8890	0.778	0.85	0.737	0.876	0.757	train
resnet50	0.75	0.7024–0.7986	0.745	0.6374–0.8535	0.818	0.709	0.628	0.867	0.711	val
resnet101	0.751	0.7035–0.7997	0.785	0.7218–0.8486	0.847	0.699	0.604	0.894	0.705	train
resnet101	0.739	0.6904–0.7885	0.76	0.6605–0.8596	0.758	0.727	0.625	0.833	0.685	val
DenseNet121	0.776	0.7306–0.8228	0.825	0.7674–0.8820	0.847	0.737	0.635	0.899	0.726	train
DenseNet121	0.75	0.7028–0.7985	0.801	0.7036–0.8975	0.848	0.691	0.622	0.884	0.718	val
DenseNet169	0.863	0.8264–0.9007	0.881	0.8304–0.9324	0.833	0.88	0.789	0.907	0.811	train
DenseNet169	0.795	0.7516–0.8398	0.817	0.7147–0.9184	0.848	0.764	0.683	0.894	0.757	val

Performance metrics for five deep learning architectures (ShuffleNet, ResNet50, ResNet101, DenseNet121, DenseNet169). DenseNet169 demonstrated the highest AUC in the validation set.

### 3.5 Transformer fusion model performance and comparison

**Figure 3:**
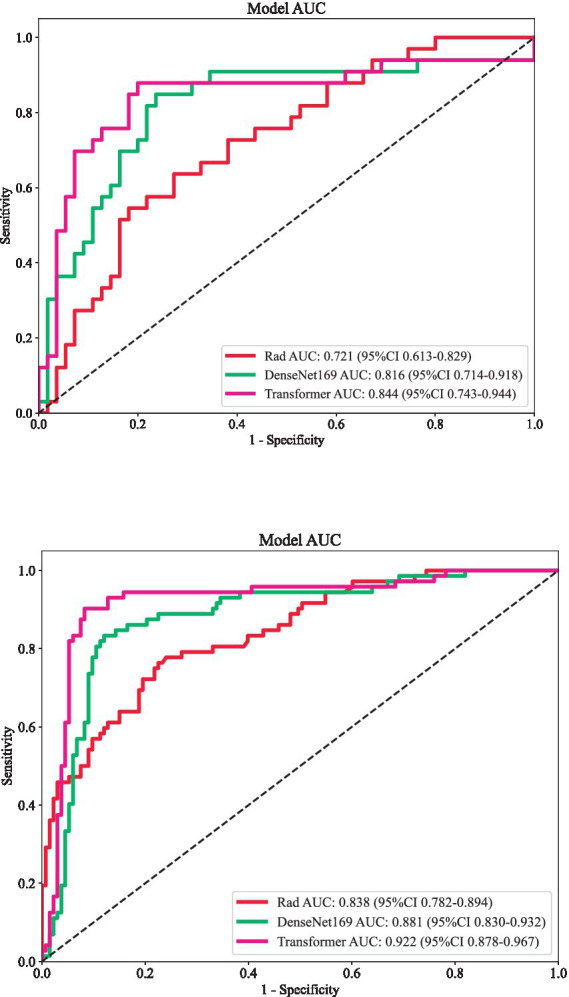
Comparison of ROC curves among optimal models. ROC comparison of the optimal radiomics-habitat model (Rad, red), the best deep learning model (DenseNet169, green), and the proposed transformer fusion model (magenta). The transformer model achieved the highest AUC (0.922 in training, 0.844 in validation), significantly outperforming the single-modality models.

The Transformer fusion model achieved: Training set Accuracy = 0.912, AUC = 0.922 (95% CI: 0.878–0.967), Sensitivity = 0.903, Specificity = 0.917, F1 = 0.878; Validation set Accuracy = 0.830, AUC = 0.844 (95% CI: 0.743–0.944), Sensitivity = 0.879, Specificity = 0.800, F1 = 0.795. The DeLong test showed that the validation AUC of the Transformer fusion model was significantly higher than that of the Radiomics-Habitat model (*p* < 0.001) and the optimal deep learning model (DenseNet169, *p* = 0.021). Compared to the DenseNet169 model, the Transformer fusion model improved validation AUC by 0.028, Specificity by 0.036, and F1 by 0.038; compared to the Radiomics-Habitat model, AUC improved by 0.117, Sensitivity by 0.121, showing significant advantages in comprehensive performance (Table 4; Figure 3). As a supplementary experiment, we further excluded 4 cases with asymptomatic morphological instability, and repeated all model evaluations in the refined cohort to verify the robustness of the diagnostic performance.

**Table 4:** Comparison of the transformer model with optimal baseline models.

Model name	Accuracy	Accuracy 95%CI	AUC	AUC 95%CI	Sensitivity	Specificity	PPV	NPV	F1	Task
Rad	0.771	0.7254–0.8176	0.838	0.7815–0.8939	0.764	0.774	0.647	0.858	0.701	Train
DenseNet169	0.863	0.8267–0.9008	0.881	0.8300–0.9323	0.833	0.88	0.789	0.907	0.811	Train
Transformer	0.912	0.8805–0.9449	0.922	0.8777–0.9667	0.903	0.917	0.855	0.946	0.878	Train
Rad	0.693	0.6375–0.7498	0.721	0.6131–0.8293	0.636	0.727	0.583	0.769	0.609	Val
DenseNet169	0.795	0.7518–0.8396	0.816	0.7137–0.9183	0.848	0.764	0.683	0.894	0.757	Val
Transformer	0.83	0.7904–0.8707	0.844	0.7428–0.9443	0.879	0.8	0.725	0.917	0.795	Val

A comparative summary of the optimal Radiomics-Habitat model (Rad), the best Deep Learning model (DenseNet169), and the proposed transformer fusion model.

### 3.6 Clinical utility assessment

**Figure 4:**
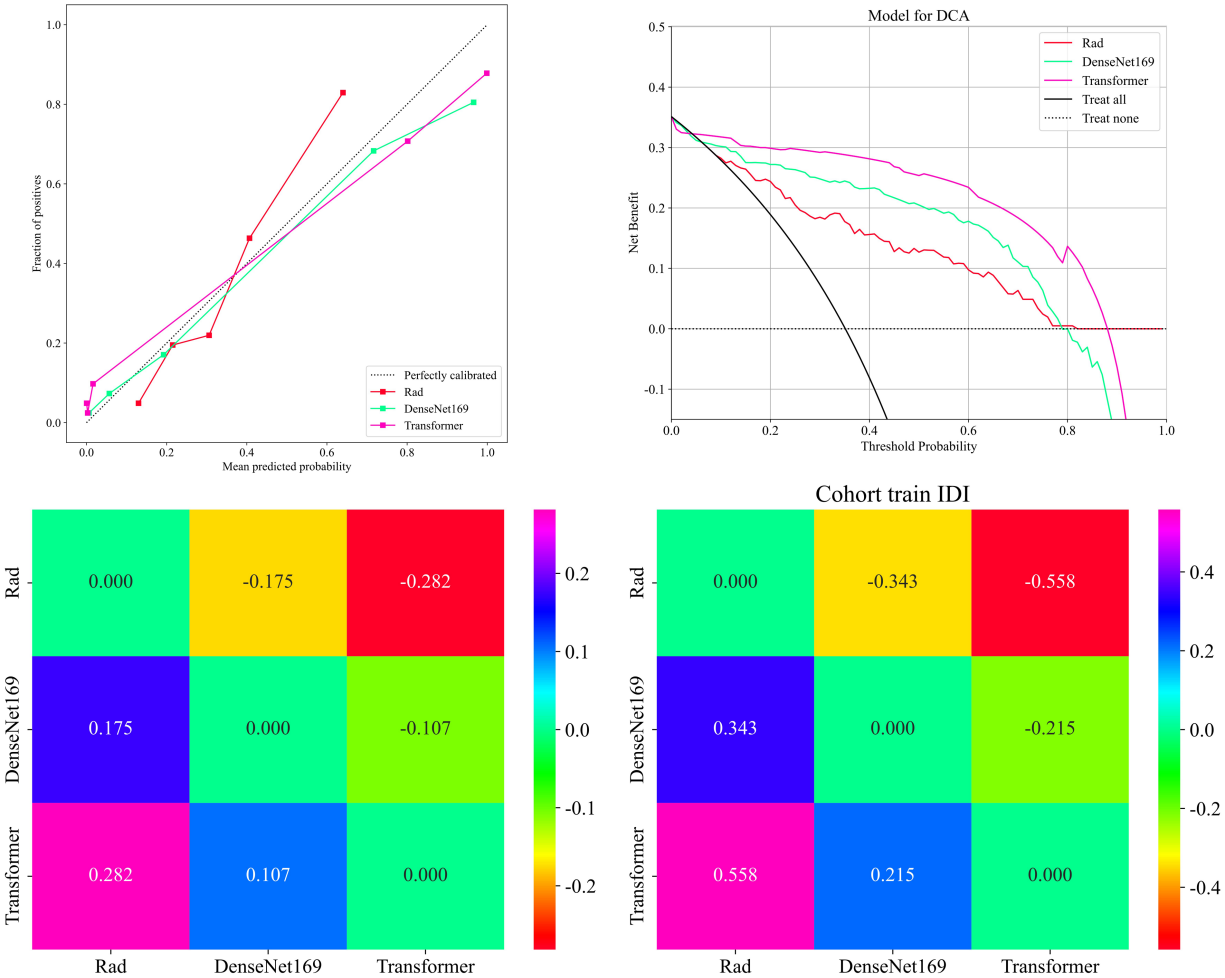
Clinical utility and decision curve analysis. (Top right) Decision curve analysis (DCA) showing the net benefit of the transformer model (magenta) is consistently higher than the Rad (red) and DenseNet169 (green) models across a wide range of threshold probabilities (0.1–0.8). (Top left) Calibration curves demonstrate that the transformer model (magenta) has the highest agreement between predicted probabilities and observed outcomes (closest to the diagonal ideal line). (Bottom) Heatmaps for net reclassification improvement (NRI) and integrated discrimination improvement (IDI) in the training cohort, indicating positive improvement for the transformer model compared to baselines.

Calibration curves showed the Transformer model achieved the highest consistency with actual outcomes (slope = 0.97). DCA confirmed its superior net benefit over other models and baseline strategies across threshold probabilities of 0.1–0.8. Positive NRI and IDI values further demonstrated that the Transformer model significantly improved predictive discrimination and clinical decision-making compared to both Radiomics-Habitat and DenseNet169 models (Figure 4). The Hosmer-Lemeshow test demonstrated calibration for the Rad model (*χ*^2^ = 17.84, *p* = 0.022) and excellent calibration for both the DenseNet169 model (*χ*^2^ = 5.12, *p* = 0.745) and the Transformer model (*χ*^2^ = 3.67, *p* = 0.885).

### 3.7 Key feature identification

**Figure 5:**
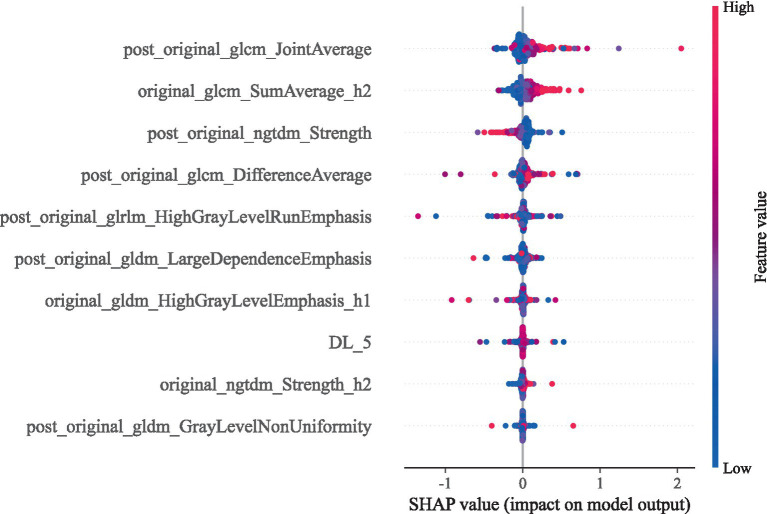
SHAP analysis of the transformer model. (Left) Feature importance ranking showing the top predictors. (Right) SHAP summary plot. Each dot represents a sample; color indicates feature value (red = high, blue = low). Features such as post_original_glcm_JointAverage and habitat-derived metrics significantly impact the model output.

SHAP analysis showed that key features affecting aneurysm stability assessment included: post_original_glrlm_HighGrayLevelRunEmphasis from GLRLM, original_gldm_HighGrayLevelEmphasis_h1 based on Habitat 1 from GLDM, post_original_gldm_GrayLevelNonUniformity and post_original_gldm_LargeDependenceEmphasis, original_glcm_SumAverage_h2 based on Habitat 2 from GLCM, post_original_glcm_JointAverage and post_original_glcm_DifferenceAverage, original_ngtdm_Strength_h2 based on Habitat 2 and post_original_ngtdm_Strength from NGTDM, as well as the high-dimensional feature DL_5 extracted by deep learning. The distribution of SHAP values shows that while the directional impact of these features on model output fluctuates, they are all core contributing factors to model decision-making (Figure 5). The distribution of these features between different groups, as well as the detailed radiomic definitions and plain-language clinical interpretations of the top 10 predictive features from the SHAP analysis, are provided in the Supplementary material.

## 4 Discussion

Accurately assessing UIA instability is crucial for optimizing treatment and preventing subarachnoid hemorrhage. Our study demonstrates that a Transformer-based fusion model significantly outperforms standalone Radiomics-Habitat and Deep Learning approaches, offering superior predictive performance and clinical utility by effectively integrating multi-dimensional feature sets from vessel wall MRI.

Evidence from existing long-term follow-up and morphological studies suggests that Aneurysm Wall Enhancement (AWE) likely serves as a direct imaging manifestation of increased endothelial permeability, neovascularization, and inflammatory interstitial edema within the vessel wall. This state of active inflammation may play a pivotal role in driving collagen remodeling and the three-dimensional morphological evolution of the aneurysm, thereby elevating the long-term risk of growth and rupture. Our experimental results, which demonstrated statistically significant differences in AWEP between stable and unstable.

aneurysm groups, corroborate this theoretical framework (Zhong et al., 2021; Liu et al., 2025; van der Kamp et al., 2025; Molenberg et al., 2021; Vergouwen et al., 2019). Concurrently, our study identified that aspirin usage and elevated blood lipid levels also exhibited significant discriminative value between the two groups. This observation is further supported by a recent study investigating the impact of statins on aneurysm wall enhancement (Zhang et al., 2025).

**Figure 6:**
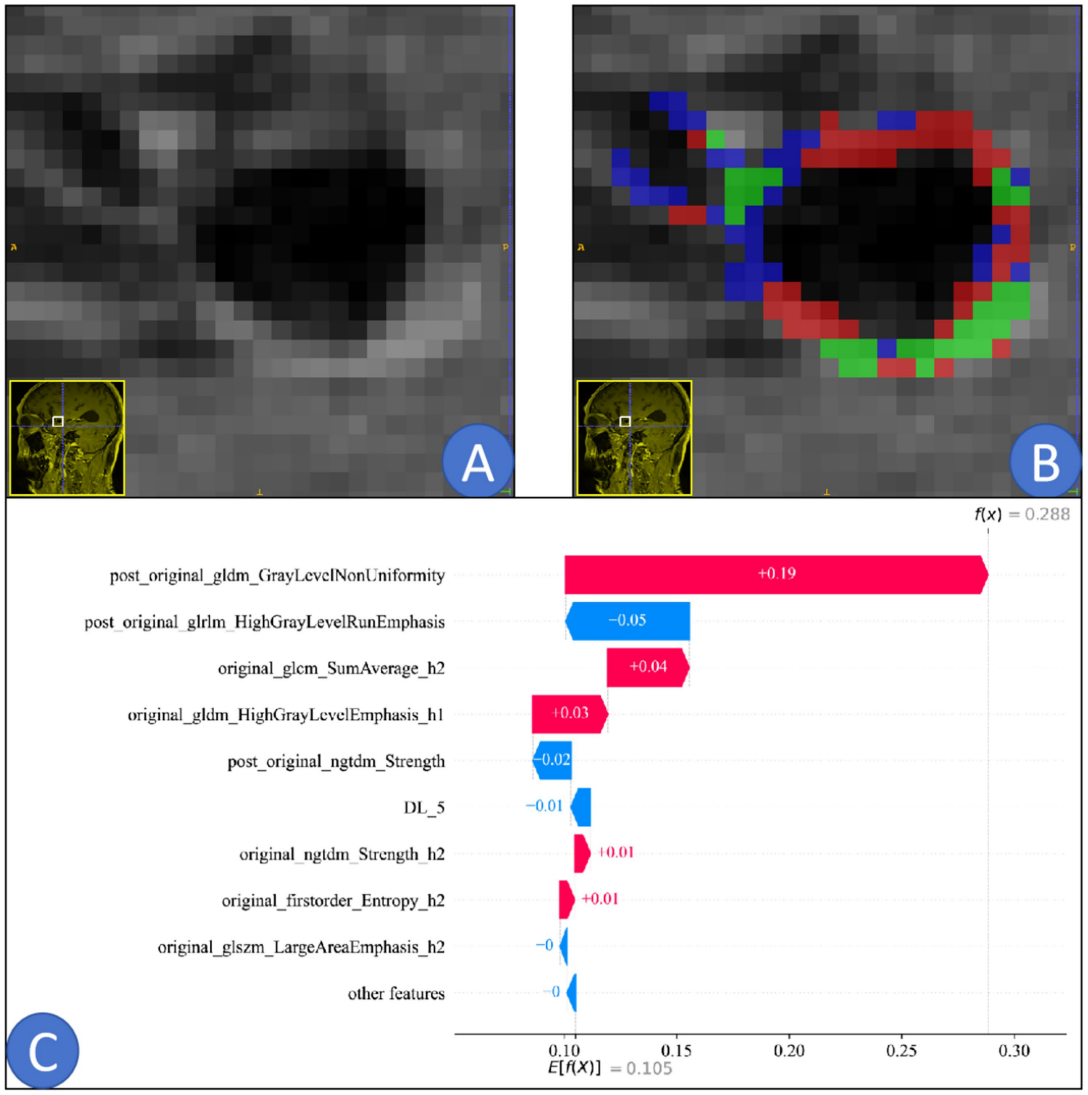
Representative case study. **(A)** Post-contrast 3D-SPACE image of a 68-year-old male with a right middle cerebral artery aneurysm (max diameter 11.6 mm, AWEP score 1). **(B)** Automatic habitat segmentation showing three subregions: Habitat 1 (red), Habitat 2 (green), and Habitat 3 (blue). **(C)** SHAP force plot for this patient from the Transformer model. Red bars indicate features pushing the prediction towards instability (positive impact), while blue bars indicate negative impact. The model correctly classified this aneurysm as unstable.

Traditional radiomics typically conceptualizes lesions as homogeneous entities, which may fail to capture the complex spatial heterogeneity in structural composition and biological behavior. We attempt to explore this limitation by employing this habitat-based fusion strategy. By partitioning ROIs into three subregions via K-means clustering, our approach successfully integrated global imaging phenotypes with localized subregional characteristics. These three habitat subregions were radiologically interpreted as distinct tissue components within and around the aneurysm on VWI. Habitat 1 may represent the non-enhancing part of the aneurysm wall, which is likely to reflect relatively stable wall tissue without obvious inflammatory changes. Habitat 2 is considered the contrast-enhancing portion of the aneurysm wall, which we speculate is associated with inflammatory infiltration of the vessel wall and corresponds well with higher AWEP grades. Habitat 3 mainly involves the parent artery and adjacent perivascular tissues, which serves as a normal vascular reference to reduce interference from non-aneurysmal signals. These findings suggest that habitat analysis may help depict the heterogeneous imaging phenotypes of UIAs, and texture features from the enhancing subregion may be important contributors to the model’s predictive performance (Figure 6).

Deep learning models offer distinct advantages through automated, high-dimensional feature extraction (Qu et al., 2024; Li et al., 2024; Shen et al., 2024). In this study, the dual-channel DenseNet169 architecture achieved a validation AUC of 0.816, outperforming the Radiomics-Habitat model. Its dense connection design facilitates feature reuse, enabling the capture of subtle imaging nuances that may elude visual inspection. However, standalone DL models often lack interpretability, which can hinder clinical translation (Növer et al., 2025).

To bridge this gap, our Transformer model (validation AUC 0.844) utilized a self-attention mechanism to model high-order correlations across clinical, radiomics-habitat, and DL features (Zhang et al., 2025). SHAP analysis sheds light on the relative importance of these high-dimensional interactions, laying a foundation for interpreting their clinical relevance. The predominant contribution of habitat-related texture features indicated by SHAP values may reflect that the model tends to capture imaging patterns from contrast-enhancing aneurysm wall subregions. Combined with calibration curves, DCA, NRI, and IDI, this framework confirms a superior net clinical benefit. Clinically, the positive net reclassification index (NRI = 0.282) indicated that the Transformer fusion model optimized the risk stratification decision for 28.2% of patients with UIAs. Meanwhile, the integrated discrimination improvement (IDI = 0.558) demonstrated a significant enhancement in the model’s discriminatory ability for distinguishing between stable and unstable aneurysms, further confirming the incremental clinical value of the multimodal fusion strategy.

Our study has certain limitations. First, it is a single-center retrospective design with a limited sample size, and it lacks an independent external validation dataset. Therefore, the current framework should be interpreted as an initial exploratory initiative that provides preliminary evidence. Further integration of diverse multi-center datasets is required to fully establish its generalizability and long-term clinical utility. Second, ROIs were manually delineated; although consistency was good, subjective bias is inevitable, and future work could combine automatic or semi-automatic segmentation algorithms. Third, this study did not include hemodynamic sequences such as 4D-Flow MRI, whose potential value in fusion with vessel wall imaging and AI models remains to be further explored. Fourthly, our multimodal framework involves computationally intensive deep-learning architectures alongside high-dimensional radiomics-habitat pipelines. Given the substantial computational overhead inherent in repeatedly training deep neural networks and Transformer models, a formal optimism-corrected bootstrap resampling was not performed to evaluate potential performance shrinkage. Therefore, our primary validation results may represent an optimistic estimation of the model’s true capability. In our future research, we anticipate expanding the cohort to include independent, external datasets from multiple clinical centers, thereby further exploring the framework’s generalizability and clinical utility.

## 5 Conclusion

Our Transformer fusion model, incorporating complementary radiomics-habitat texture signatures, deep learning-extracted features, and clinical variables, presents an exploratory and interpretable risk-assessment framework for evaluating UIA instability. While it demonstrates potential to assist clinicians in optimizing individualized aneurysm management strategies, it serves strictly as a hypothesis-generating pilot study and requires future large-scale, prospective multi-center validation before any clinical translation.

## Data Availability

The raw data supporting the conclusions of this article will be made available by the authors, without undue reservation.

## References

[ref1] Benndorf G. (2023). Advancing vessel wall imaging in intracranial aneurysms: a crucial step towards improved patient management? Acta Neurochir. 165, 3831–3832. doi: 10.1007/s00701-023-05773-6 37861925

[ref2] Dier C. Sanchez S. Sagues E. Gudino A. Jaramillo R. Wendt L. . (2025). Radiomic profiling of high-risk aneurysms with blebs: an exploratory study. Journal of neurointerventional surgery 17, 1096–1101. doi: 10.1136/jnis-2024-022133 39299742

[ref3] Edjlali M. Gentric J. C. Régent-Rodriguez C. Trystram D. Hassen W. B. Lion S. . (2014). Does aneurysmal wall enhancement on vessel wall MRI help to distinguish stable from unstable intracranial aneurysms? Stroke 45, 3704–3706. doi: 10.1161/STROKEAHA.114.006364 25325912

[ref4] Gatenby R. A. Grove O. Gillies R. J. (2013). Quantitative imaging in cancer evolution and ecology. Radiology 269, 8–14. doi: 10.1148/radiol.13122697, 24062559 PMC3781355

[ref5] Laukka D. Kivelev J. Rahi M. Vahlberg T. Paturi J. Rinne J. . (2024). Detection rates and trends of asymptomatic unruptured intracranial aneurysms. Neurosurgery 94, 297–306. doi: 10.1227/neu.0000000000002408 37695560 PMC10766300

[ref6] Li Y. Zhang H. Sun Y. Fan Q. Wang L. Ji C. . (2024). Deep learning-based platform for intracranial aneurysms detection in 3D brain TOF-MRA. Int. J. Med. Inform. 188:105487. doi: 10.1016/j.ijmedinf.2024.105487 38761459

[ref7] Liu Q. Jiang P. Jiang Y. Ge H. Li S. Jin H. . (2021). Development and validation of an institutional nomogram for aneurysm rupture risk stratification. Sci. Rep. 11:13826. doi: 10.1038/s41598-021-93347-5 34226632 PMC8257713

[ref8] Liu Q. Nie X. Vergouwen M. D. I. Wang Y. He H. Wu J. . (2025). Gadolinium-enhanced aneurysm wall imaging and risk of intracranial aneurysm growth or rupture. JAMA Neurol. 82, 1135–1143. doi: 10.1001/jamaneurol.2025.2432 40920406 PMC12418222

[ref9] Long H. Che W. Yang C. Liao Y. Wu J. Chen C. . (2025). Identification of key risk factors for rupture in small intracranial aneurysms. World Neurosurg. 194:123552. doi: 10.1016/j.wneu.2024.123552 39653080

[ref10] Molenberg R. Aalbers M. W. Appelman A. P. A. Uyttenboogaart M. van Dijk J. M. C. . (2021). Intracranial aneurysm wall enhancement as an indicator of instability: a systematic review and meta-analysis.Eur. J. Neurol. 28, 2259–2268. doi: 10.1111/ene.14761 PMC9292155 34424585

[ref11] Növer M. Styczen H. Jabbarli R. Dammann P. Köhrmann M. Hagenacker T. . (2025). Prediction of recurrence and rupture risk of intracranial aneurysms of the posterior circulation. Diagnostics 15:2365. doi: 10.3390/diagnostics15182365 41008736 PMC12468337

[ref12] Qu J. Niu H. Li Y. Chen T. Peng F. Xia J. . (2024). A deep learning framework for intracranial aneurysms automatic segmentation and detection. Eur. Radiol. 34, 2838–2848. doi: 10.1007/s00330-023-09934-5 37843574

[ref13] Samaniego E. A. Roa J. A. Hasan D. (2019). Vessel wall imaging in intracranial aneurysms. J NeuroInterv Surg 11, 1105–1112. doi: 10.1136/neurintsurg-2019-014318 31337731

[ref14] Santarosa C Cord B Koo A Bhogal P Malhotra A Payabvash S . Vessel wall magnetic resonance imaging in intracranial aneurysms: principles and emerging clinical applications. Interv. Neuroradiol. (2020). 26:135–146. doi: 10.1177/1591019919891297 31818175 PMC7507220

[ref15] Shen Y. Zhu C. Chu B. Song J. Geng Y. Li J. . (2024). Evaluation of the clinical application value of artificial intelligence in diagnosing head and neck aneurysms. BMC Med. Imaging 24:261. doi: 10.1186/s12880-024-01813-4 39354383 PMC11446065

[ref16] Society of Neurosurgery of Chinese Medical Association (2024). Chinese guideline for the clinical management of patients with unruptured intracranial aneurysms (2024). Chin Med J (in Chinese) 104, 1918–1939. doi: 10.3760/cma.j.issn.0376-2491.2024.25.002 38825938

[ref17] Sohrabi-Ashlaghi A. Azizi N. Abbastabar H. Shakiba M. Zebardast J. Firouznia K. . (2024). Accuracy of radiomics-based models in distinguishing ruptured and unruptured intracranial aneurysms. Eur. J. Radiol. 181:111739. doi: 10.1016/j.ejrad.2024.111739 39293240

[ref18] Thompson B. G. Brown R. D. Jr. Amin-Hanjani S. Broderick J. P. Cockroft K. M. Connolly E. S. Jr. . (2015). Guidelines for the management of patients with unruptured intracranial aneurysms. Stroke 46, 2368–2400. doi: 10.1161/STR.0000000000000069 26089327

[ref19] Turhon M. Li M. Kang H. Huang J. Zhang F. Zhang Y. . (2023). Development and validation of a deep learning model for prediction of intracranial aneurysm rupture risk based on multi-omics factor. Eur. Radiol. 33, 6759–6770. doi: 10.1007/s00330-023-09672-3, 37099175

[ref20] van der Kamp L. T. van der Schaaf I. C. Edjlali M. Naggara O. Matsushige T. Bulters D. O. . (2025). Appearance and disappearance of intracranial Aneurysm Wall enhancement during follow-up: a Multicenter cohort study. AJNR Am. J. Neuroradiol. 46, 2479–2484. doi: 10.3174/ajnr.A8906 41233156 PMC12687981

[ref21] Vergouwen M. D. I. Backes D. van der Schaaf I. C. Hendrikse J. Kleinloog R. Algra A. . (2019). Gadolinium enhancement of the aneurysm wall in unruptured intracranial aneurysms is associated with an increased risk of aneurysm instability: a follow-up study. AJNR Am. J. Neuroradiol. 40, 1112–1116. doi: 10.3174/ajnr.A6105 31221634 PMC7048551

[ref22] Yan C. Zhang H. Zhang X. Wang K. Yang M. Wang F. . (2025). The roles of radiomics and deep learning for automatic detection, stability assessment, and rupture risk prediction in intracranial aneurysms: a systematic review. Eur. J. Med. Res. doi: 10.1186/s40001-025-03588-y PMC12777297 41327324

[ref23] Zhang H. Lu C. Hu Z. Sun D. Li L. Wu H. . (2025). Prediction and SHAP analysis integrating morphological and hemodynamic parameters for Unruptured intracranial aneurysm occlusion after flow diverter treatment. CNS Neurosci. Ther. 31:e70386. doi: 10.1111/cns.14321 40237244 PMC12001072

[ref24] Zhang X. Shen Y. Y. Su G. H. Guo Y. Zheng R. C. Du S. Y. . (2025). A dynamic contrast-enhanced MRI-based vision transformer model for distinguishing HER2 expression in breast cancer. Adv Sci (Weinh) 12:e03925. doi: 10.1002/advs.202403925 40488332 PMC12412478

[ref25] Zhang Y. Wang C. Dong L. Turhon M. Kang H. Liu J. . (2025). Statins reduce wall enhancement at vessel wall MRI in unruptured vertebrobasilar dissecting aneurysms: a randomized controlled trial. Radiology 317:e242806. doi: 10.1148/radiol.242806 41432563 PMC13267880

[ref26] Zhong W. Su W. Li T. Tan X. Chen C. Wang Q. . (2021). Aneurysm wall enhancement in unruptured intracranial aneurysms: a histopathological evaluation. J. Am. Heart Assoc. 10:e018633. doi: 10.1161/JAHA.120.018633, 33410330 PMC7955308

[ref27] Zhou J. Mou Q. Song Y. Wu Y. Liu M. . (2025). Comparative analysis and influencing factors of aneurysm wall characteristics based on artificial intelligence-assisted 3.0T MRI and 5.0T MRI high-resolution vascular wall imaging. BMC Med. Imaging. doi: 10.1186/s12880-025-01959-9 PMC12781677 41339837

