# Supplementary material for: Transformer-based fusion of radiomics-habitat and deep learning for assessing unruptured intracranial aneurysm instability
Source: Front Neurosci. 2026 Jun 17;20:1818110. doi: 10.3389/fnins.2026.1818110 (PMC13318966; doi:10.3389/fnins.2026.1818110)
Supplement: Supplementary file 1 [file Data_Sheet_1.docx]

1. **Specific parameters for MRI scanning**

SPACE T1-weighted sequence: TR/TE: 900/15 ms, Field of View (FOV) = 200x200 mm², Voxel size: 0.5x0.5x0.5 mm³, 224 slices, scan time: 7 min 36 s. For contrast-enhanced scanning, gadopentetate dimeglumine (0.1 mmol/kg) was injected via the cubital vein at a rate of 2.0 ml/s, with scanning initiating 10 minutes post-injection. 3D-TOF MRA was used to localize the aneurysm, clarify the anatomy of the parent artery, and assess the overall vascular condition.

1. Pretrained Model Initialization

All convolutional neural network models used in this study, including ResNet50, ResNet101, DenseNet121, DenseNet169, and ShuffleNet, were initialized with publicly available pretrained weights provided by the PyTorch torchvision library. The pretrained weights were obtained by training the backbone networks on the ImageNet-1K dataset (ILSVRC 2012.

In our experiments, these pretrained weights were adopted for model initialization to leverage transferable visual features learned from large‑scale data. This strategy effectively accelerated model convergence and enhanced generalization performance on our target dataset. The network architectures and pretrained weight configurations strictly followed the official implementation of torchvision (version:0.15.2). No additional self‑supervised pretraining or custom weight pretraining was conducted in this work.

## Baseline clinical characteristics of patients

This section presents patient-level baseline characteristics of the study cohort. Two tables are provided:Table 1 shows comparisons of demographic and clinical variables between patients with stable and unstable aneurysms in the training and validation sets, respectively. Table 2 presents the overall comparison of baseline characteristics between the training and validation sets to verify the randomness and comparability of dataset splitting..

| **feature_name** | **Train dataset** | | |  | **Val dataset** | | |  |
| --- | --- | --- | --- | --- | --- | --- | --- | --- |
|  | **ALL（205）** | **stable(133)** | **unstable(72)** | ***p*** | **ALL(88)** | **stable(55)** | **unstable(33)** | ***p*** |
| size | 6.43(3.80,8.60) | 6.03(3.27,7.91) | 7.19(4.21,9.34) | 0.045 | 6.65(3.49,8.13) | 6.14(3.26,7.85) | 6.79(3.60,8.76) | 0.928 |
| age | 56.91±9.09 | 56.59±9.61 | 57.49±7.92 | 0.818 | 57.27±8.76 | 58.67±9.38 | 54.94±8.12 | 0.116 |
| location |  |  |  | 0.631 |  |  |  | 0.135 |
| 0 | 123(60.00) | 83(62.41) | 40(55.56) |  | 53(60.23) | 35(63.64) | 18(54.55) |  |
| 2 | 44(21.46) | 27(20.30) | 17(23.61) |  | 20(22.73) | 14(25.45) | 6(18.18) |  |
| 4 | 38(18.54) | 23(17.29) | 15(20.83) |  | 15(17.05) | 6(10.91) | 9(27.27) |  |
| AWEP |  |  |  | <0.001 |  |  |  | 0.015 |
| 0 | 122(59.51) | 90(67.67) | 32(44.44) |  | 45(51.14) | 32(58.18) | 13(39.39) |  |
| 1 | 69(33.66) | 39(29.32) | 30(41.67) |  | 36(40.91) | 22(40.00) | 14(42.42) |  |
| 2 | 14(6.83) | 4(3.01) | 10(13.89) |  | 7(7.95) | 1(1.82) | 6(18.18) |  |
| hypertension |  |  |  | 1 |  |  |  | 0.699 |
| 0 | 78(38.05) | 51(38.35) | 27(37.50) |  | 47(53.41) | 28(50.91) | 19(57.58) |  |
| 1 | 127(61.95) | 82(61.65) | 45(62.50) |  | 41(46.59) | 27(49.09) | 14(42.42) |  |
| hyperlipidemia |  |  |  | 0.012 |  |  |  | 0.044 |
| 0 | 151(73.66) | 106(79.70) | 45(62.50) |  | 63(71.59) | 44(80.00) | 19(57.58) |  |
| 1 | 54(26.34) | 27(20.30) | 27(37.50) |  | 25(28.41) | 11(20.00) | 14(42.42) |  |
| diabetes |  |  |  | 1 |  |  |  | 0.862 |
| 0 | 176(85.85) | 114(85.71) | 62(86.11) |  | 78(88.64) | 48(87.27) | 30(90.91) |  |
| 1 | 29(14.15) | 19(14.29) | 10(13.89) |  | 10(11.36) | 7(12.73) | 3(9.09) |  |
| cerebral_infarction_history |  |  |  | 0.833 |  |  |  | 0.626 |
| 0 | 140(68.29) | 92(69.17) | 48(66.67) |  | 71(80.68) | 43(78.18) | 28(84.85) |  |
| 1 | 65(31.71) | 41(30.83) | 24(33.33) |  | 17(19.32) | 12(21.82) | 5(15.15) |  |
| SAH_history |  |  |  | 0.733 |  |  |  | 1 |
| 0 | 199(97.07) | 130(97.74) | 69(95.83) |  | 83(94.32) | 52(94.55) | 31(93.94) |  |
| 1 | 6(2.93) | 3(2.26) | 3(4.17) |  | 5(5.68) | 3(5.45) | 2(6.06) |  |
| smoke |  |  |  | 0.219 |  |  |  | 1 |
| 0 | 172(83.90) | 108(81.20) | 64(88.89) |  | 75(85.23) | 47(85.45) | 28(84.85) |  |
| 1 | 33(16.10) | 25(18.80) | 8(11.11) |  | 13(14.77) | 8(14.55) | 5(15.15) |  |
| alchol |  |  |  | 1 |  |  |  | 1 |
| 0 | 183(89.27) | 119(89.47) | 64(88.89) |  | 76(86.36) | 48(87.27) | 28(84.85) |  |
| 1 | 22(10.73) | 14(10.53) | 8(11.11) |  | 12(13.64) | 7(12.73) | 5(15.15) |  |
| family_history |  |  |  | 0.587 |  |  |  | 0.095 |
| 0 | 202(98.54) | 132(99.25) | 70(97.22) |  | 85(96.59) | 55(100.00) | 30(90.91) |  |
| 1 | 3(1.46) | 1(0.75) | 2(2.78) |  | 3(3.41) | null | 3(9.09) |  |
| Aspirin |  |  |  | <0.001 |  |  |  | <0.001 |
| 0 | 64(31.22) | 28(21.05) | 36(50.00) |  | 25(28.41) | 5(9.09) | 20(60.61) |  |
| 1 | 141(68.78) | 105(78.95) | 36(50.00) |  | 63(71.59) | 50(90.91) | 13(39.39) |  |
| gender |  |  |  | 0.917 |  |  |  | 1 |
| 0 | 122(59.51) | 80(60.15) | 42(58.33) |  | 59(67.05) | 37(67.27) | 22(66.67) |  |
| 1 | 83(40.49) | 53(39.85) | 30(41.67) |  | 29(32.95) | 18(32.73) | 11(33.33) |  |

| **feature_name** | **ALL** | **Train** | **Val** | ***p* value** |
| --- | --- | --- | --- | --- |
| size | 117.32±66.68 | 121.07±66.29 | 108.58±67.16 | 0.145 |
| age | 57.02±12.12 | 56.91±12.18 | 57.27±12.04 | 0.696 |
| location |  |  |  | 0.941 |
| 0 | 176(60.07) | 123(60.00) | 53(60.23) |  |
| 2 | 64(21.84) | 44(21.46) | 20(22.73) |  |
| 4 | 53(18.09) | 38(18.54) | 15(17.05) |  |
| AWEP |  |  |  | 0.413 |
| 0 | 167(57.00) | 122(59.51) | 45(51.14) |  |
| 1 | 105(35.84) | 69(33.66) | 36(40.91) |  |
| 2 | 21(7.17) | 14(6.83) | 7(7.95) |  |
| hypertension |  |  |  | 0.021 |
| 0 | 125(42.66) | 78(38.05) | 47(53.41) |  |
| 1 | 168(57.34) | 127(61.95) | 41(46.59) |  |
| hyperlipidemia |  |  |  | 0.824 |
| 0 | 214(73.04) | 151(73.66) | 63(71.59) |  |
| 1 | 79(26.96) | 54(26.34) | 25(28.41) |  |
| diabetes |  |  |  | 0.649 |
| 0 | 254(86.69) | 176(85.85) | 78(88.64) |  |
| 1 | 39(13.31) | 29(14.15) | 10(11.36) |  |
| cerebral_infarction_history |  |  |  | 0.043 |
| 0 | 211(72.01) | 140(68.29) | 71(80.68) |  |
| 1 | 82(27.99) | 65(31.71) | 17(19.32) |  |
| SAH_history |  |  |  | 0.423 |
| 0 | 282(96.25) | 199(97.07) | 83(94.32) |  |
| 1 | 11(3.75) | 6(2.93) | 5(5.68) |  |
| smoke |  |  |  | 0.912 |
| 0 | 247(84.30) | 172(83.90) | 75(85.23) |  |
| 1 | 46(15.70) | 33(16.10) | 13(14.77) |  |
| alchol |  |  |  | 0.608 |
| 0 | 259(88.40) | 183(89.27) | 76(86.36) |  |
| 1 | 34(11.60) | 22(10.73) | 12(13.64) |  |
| family_history |  |  |  | 0.53 |
| 0 | 287(97.95) | 202(98.54) | 85(96.59) |  |
| 1 | 6(2.05) | 3(1.46) | 3(3.41) |  |
| Aspirin |  |  |  | 0.733 |
| 0 | 89(30.38) | 64(31.22) | 25(28.41) |  |
| 1 | 204(69.62) | 141(68.78) | 63(71.59) |  |
| gender |  |  |  | 0.278 |
| 0 | 181(61.77) | 122(59.51) | 59(67.05) |  |
| 1 | 112(38.23) | 83(40.49) | 29(32.95) |  |

1. **Transformer-based Fusion Model architecture and training parameters**

Architecture: The model utilized two encoder layers with an 8-head multi-attention mechanism and a feed-forward network (hidden dimension: 256). Feature vectors were normalized to the [0, 1] range and embedded as tokens. The self-attention mechanism was employed to capture high-order correlations between these heterogeneous features.Hyperparameters: The model was trained with a batch size of 5 (validation batch size = 8) using the AdamW optimizer (initial learning rate = 1e-5; weight decay = 0.1).Regularization & Optimization: A dropout rate of 0.3 was applied to prevent overfitting. We utilized the Reduce_On_Plateau scheduler, which halved the learning rate (factor = 0.5) if the validation loss failed to improve for three consecutive epochs.Implementation: Developed using PyTorch 1.12.0, the model was initialized with pre-trained weights. The final output layer used a sigmoid function to generate the instability probability

1. **Calinski-Harabasz index, Davies-Bouldin index, and Silhouette Coefficient for each clusters**


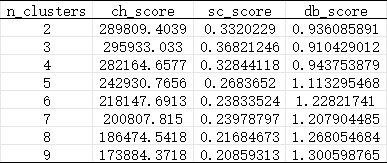


Evaluation of clustering performance for K-means algorithm with different cluster numbers.The table presents the Calinski-Harabasz index (ch_score), Silhouette Coefficient (sc_score), and Davies-Bouldin index (db_score) for cluster numbers ranging from 2 to 9. Based on the comprehensive analysis of these metrics, 3 was identified as the optimal number of clusters for the voxel-based analysis.

1. PCA dimensionality reduction of deep learning features.

To reduce feature redundancy and computational complexity, PCA was performed on the high-dimensional deep learning features. A total of 64 principal components were retained, which accounted for no less than 95% of the total variance, ensuring the integrity of the extracted imaging features.


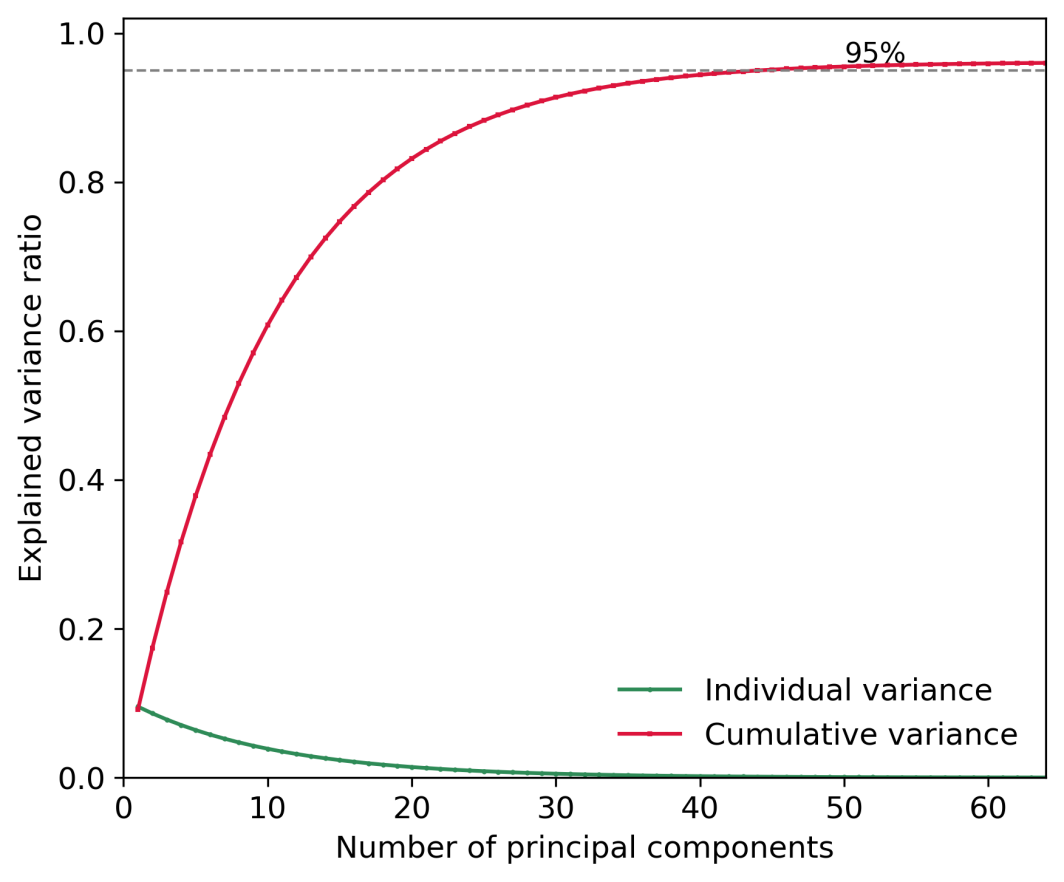


PCA dimensionality reduction of deep learning features.The 1024-dimensional features extracted from the penultimate layer of the DenseNet169 model were reduced to 64 dimensions using principal component analysis (PCA). The cumulative explained variance ratio reached ≥ 95%, indicating that the low-dimensional features retained sufficient information for subsequent modeling.

1. **Feature Weights and ROC Curves for Radiomics-Habitat Models**

Receiver Operating Characteristic (ROC) curves for all eight machine learning classifiers in the validation set are shown in Supplementary Figure 3A. The Random Forest (RF) model achieved the highest validation AUC of 0.721, demonstrating the best generalization performance among traditional machine learning algorithms. The LASSO regression coefficient plot (Supplementary Figure 3B) visualizes the magnitude and direction of the selected features' contributions to aneurysm instability prediction. After removing features with ICC < 0.80 and eliminating multicollinear features (|r| ≥ 0.90), LASSO regression with 10-fold cross-validation was applied to the radiomics-habitat feature set to identify the most predictive features for aneurysm instability. A total of 13 features were retained with non-zero regression coefficients, comprising 5 habitat-specific features (2 from Habitat 1, 2 from Habitat 2, and 1 from Habitat 3) and 8 global radiomics features.


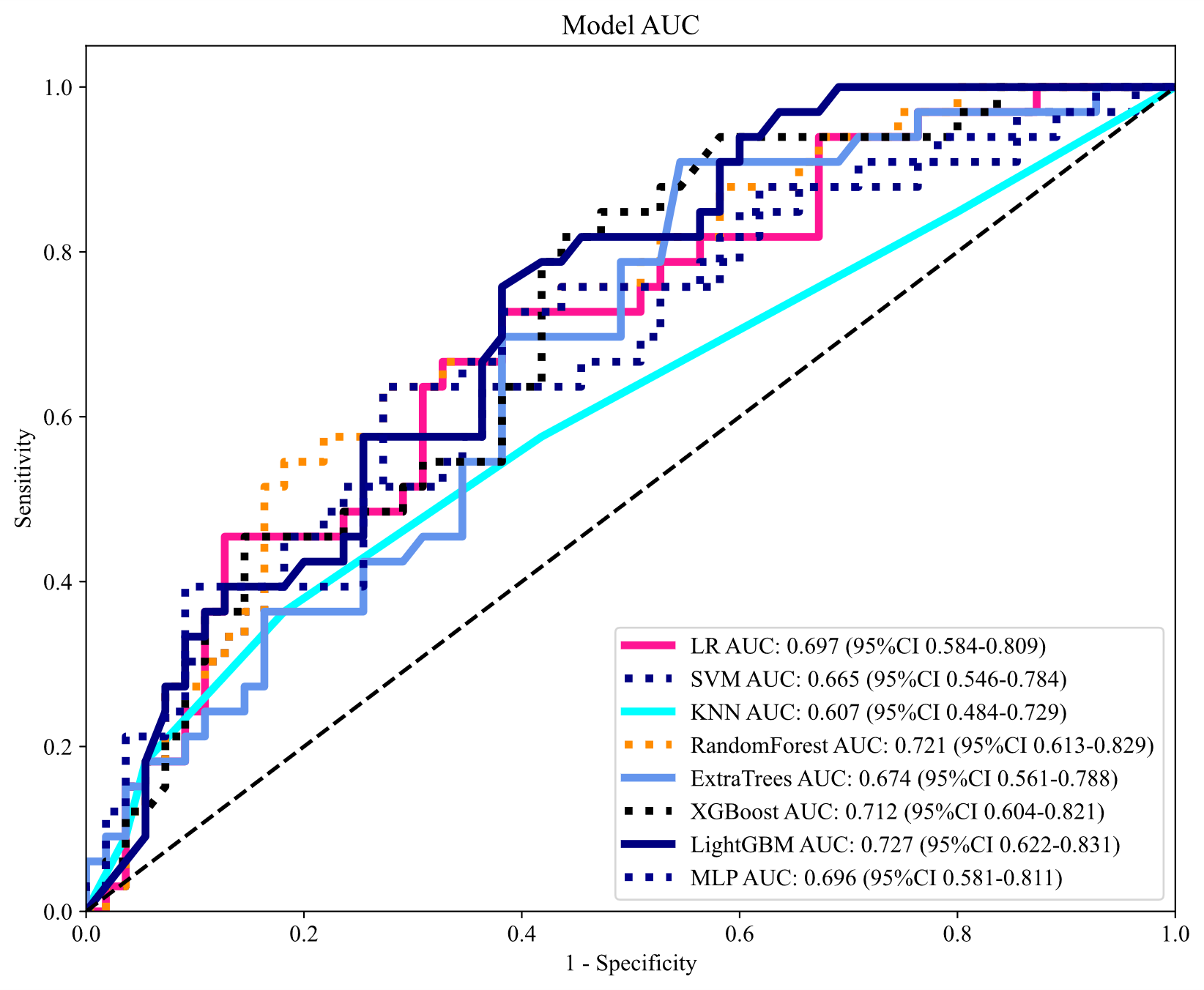

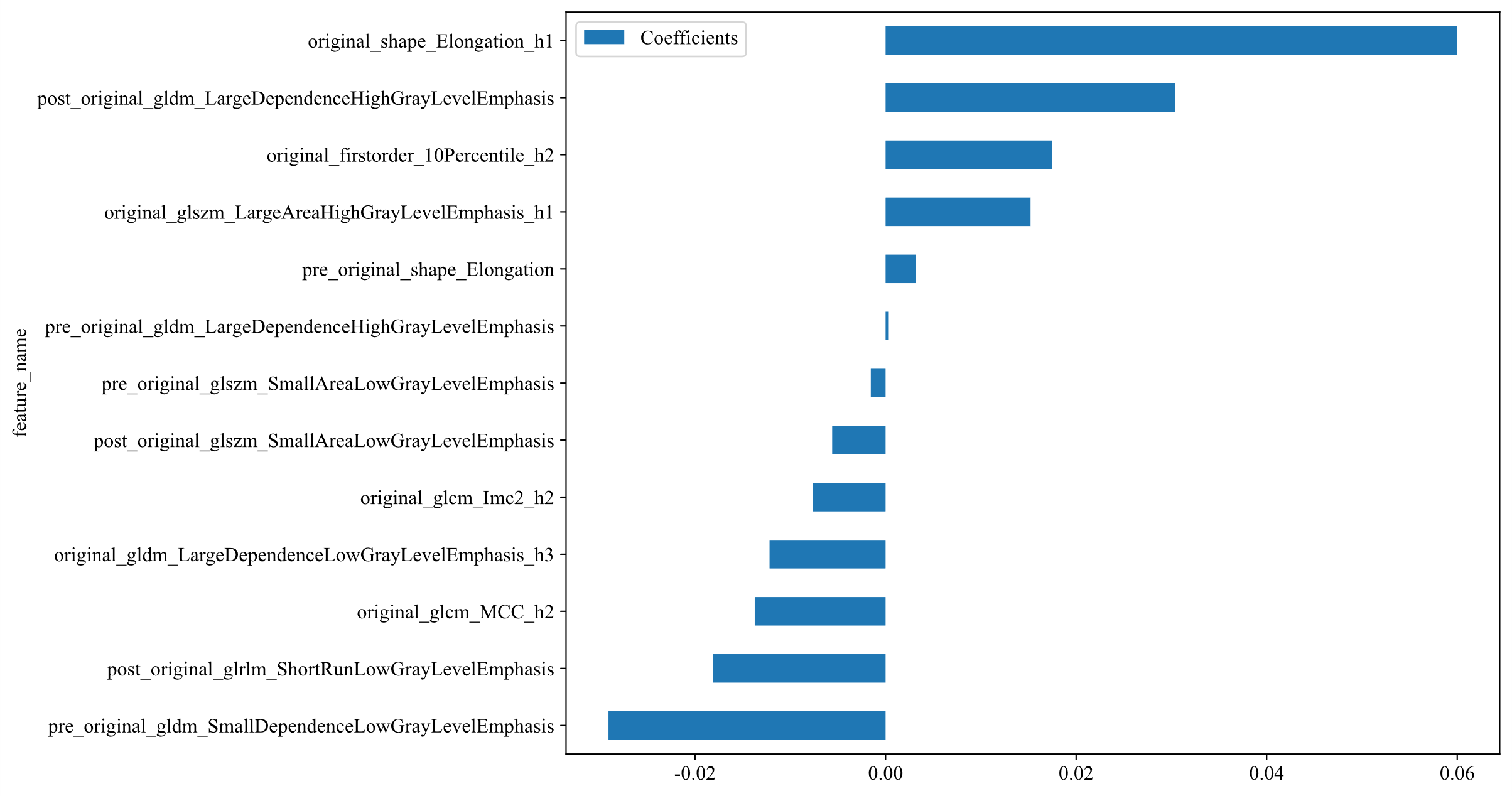
Habitat-derived features accounted for 38.5% of the selected features, highlighting the importance of capturing intralesional spatial heterogeneity in aneurysm stability assessment.

**
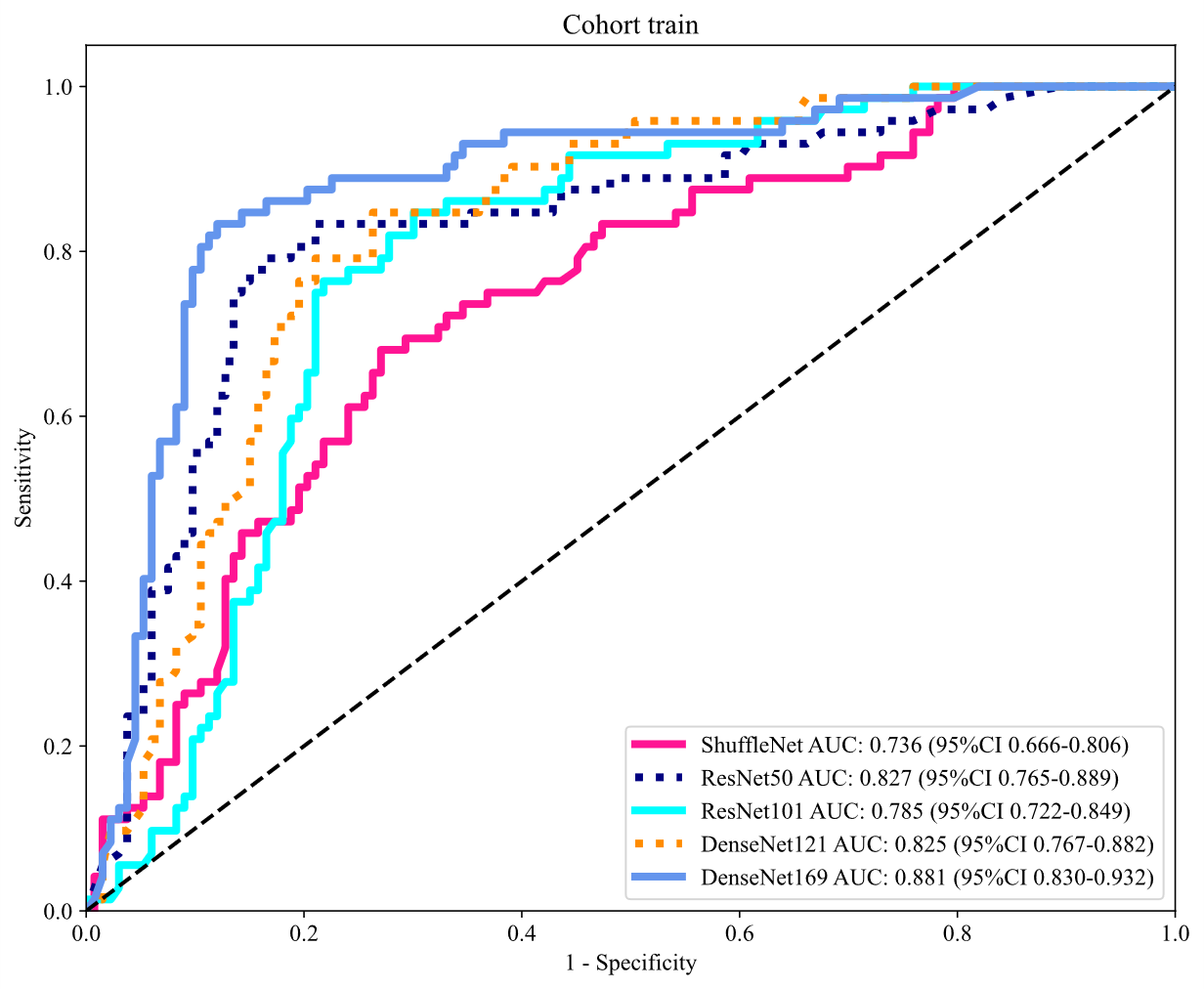
**
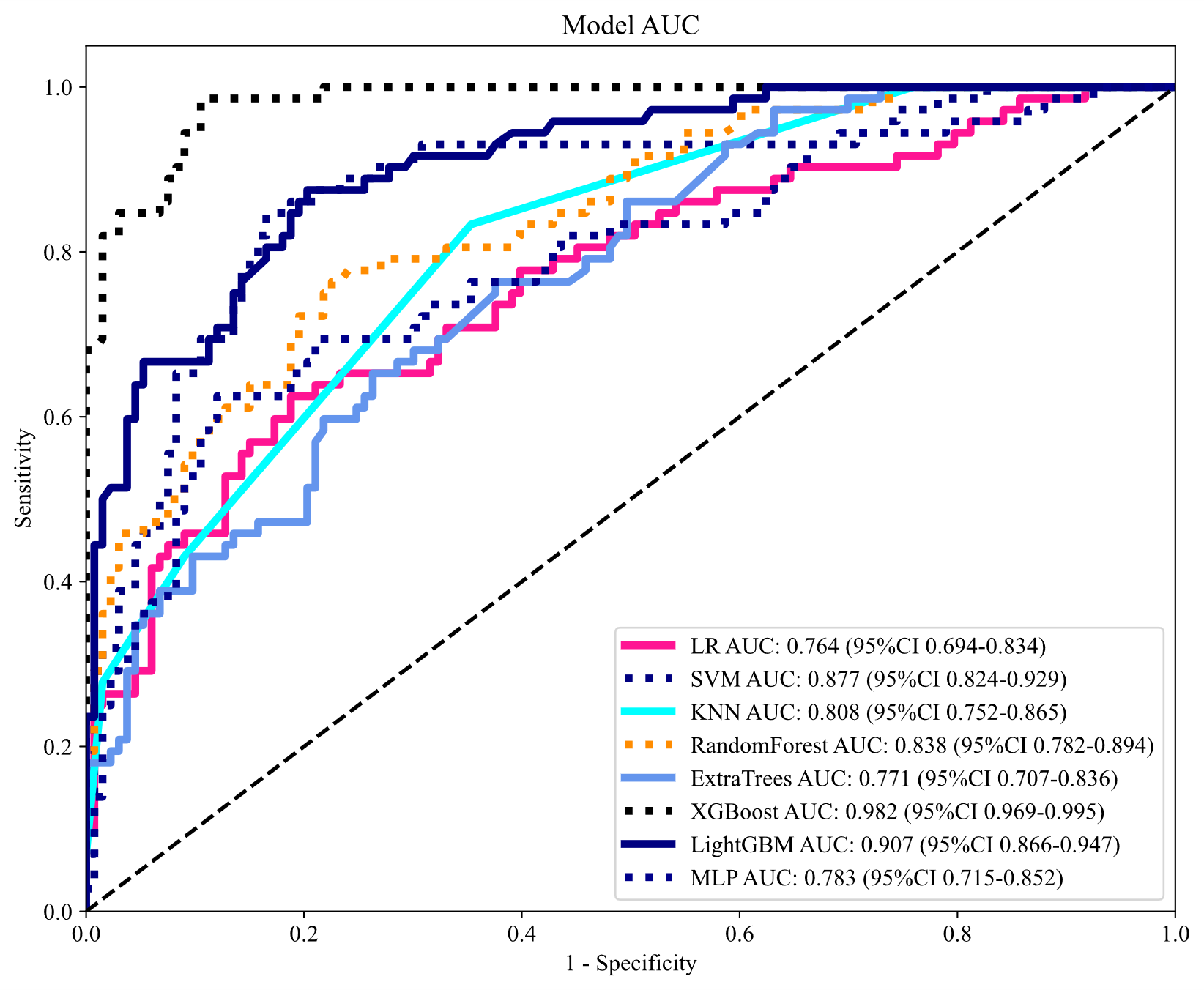


| **Rank** | **Feature Name** | **Feature Category** | **LASSO Coefficient** |
| --- | --- | --- | --- |
| 1 | pre_original_gldm_SmallDependenceLowGrayLevelEmphasis | Global | 0.062 |
| 2 | post_original_glrlm_ShortRunLowGrayLevelEmphasis | Global | 0.058 |
| 3 | original_glcm_MCC_h2 | Habitat 2 | 0.054 |
| 4 | original_gldm_LargeDependenceLowGrayLevelEmphasis_h3 | Habitat 3 | 0.051 |
| 5 | original_glcm_Imc2_h2 | Habitat 2 | 0.047 |
| 6 | post_original_glszm_SmallAreaLowGrayLevelEmphasis | Global | 0.043 |
| 7 | pre_original_glszm_SmallAreaLowGrayLevelEmphasis | Global | 0.039 |
| 8 | pre_original_gldm_LargeDependenceHighGrayLevelEmphasis | Global | 0.035 |
| 9 | pre_original_shape_Elongation | Global | 0.031 |
| 10 | original_glszm_LargeAreaHighGrayLevelEmphasis_h1 | Habitat 1 | 0.026 |
| 11 | original_firstorder_10Percentile_h2 | Habitat 2 | 0.022 |
| 12 | post_original_gldm_LargeDependenceHighGrayLevelEmphasis | Global | 0.018 |
| 13 | original_shape_Elongation_h1 | Habitat 1 | -0.012 |

1. **ROC Curves of Deep Learning Models.**

Fig. 2 ROC Curves of Deep Learning Models. Receiver Operating Characteristic curves for the five deep learning architectures in the training and validation cohorts. DenseNet169 (light blue line) achieved the highest AUC (0.817) in the validation set.

**
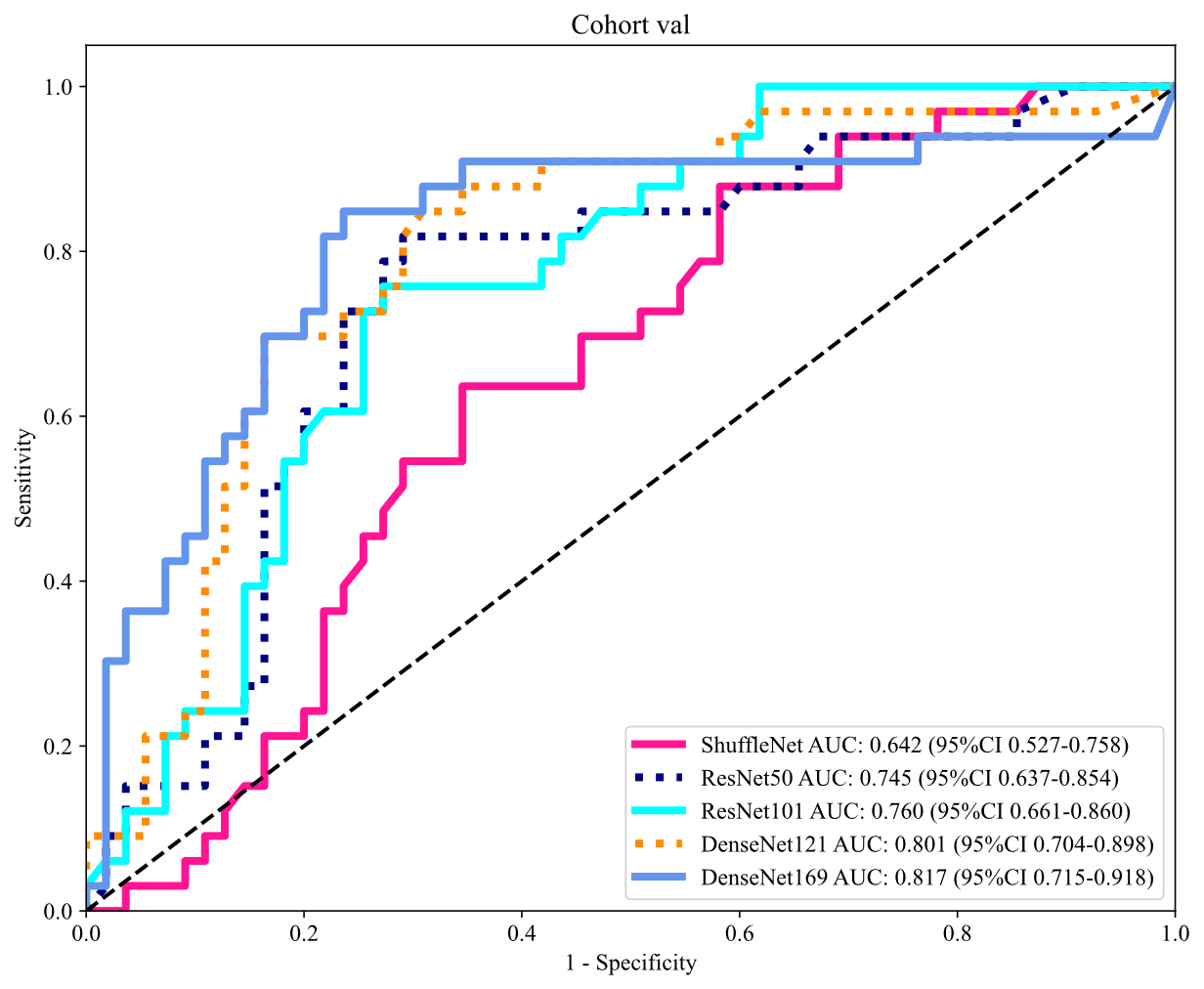
**

1. **Sensitivity Analysis After Case Exclusion**

In addition to the primary analysis, a supplementary sensitivity analysis was performed to test the robustness of the models. Four patients with asymptomatic intracranial aneurysms defined as unstable only by morphological features were excluded (2 from the training set and 2 from the validation set). All model training and validation procedures were repeated using the refined cohort, and the diagnostic performance metrics were recalculated for comparison with the primary results.

| **Modelname** | **Accuracy** | **AUC** | **95% CI** | **Sensitivity** | **Specificity** | **PPV** | **NPV** | **F1** | **Task** |
| --- | --- | --- | --- | --- | --- | --- | --- | --- | --- |
| Rad | 0.762 | 0.829 | 0.7702 - 0.8871 | 0.751 | 0.782 | 0.639 | 0.863 | 0.692 | Train |
| DenseNet169 | 0.855 | 0.872 | 0.8211 - 0.9245 | 0.821 | 0.889 | 0.781 | 0.912 | 0.802 | Train |
| Transformer | 0.904 | 0.913 | 0.8682 - 0.9591 | 0.891 | 0.925 | 0.847 | 0.951 | 0.869 | Train |
| Rad | 0.684 | 0.712 | 0.6045 - 0.8207 | 0.623 | 0.736 | 0.575 | 0.775 | 0.6 | Val |
| DenseNet169 | 0.786 | 0.807 | 0.7051 - 0.9109 | 0.835 | 0.773 | 0.675 | 0.899 | 0.748 | Val |
| Transformer | 0.821 | 0.835 | 0.7364 - 0.9372 | 0.866 | 0.809 | 0.717 | 0.922 | 0.786 | Val |

1. **Diagnostic performance of all prediction models in the refined cohort**

To verify the robustness of the predictive models for intracranial aneurysm instability, we performed a supplementary analysis by excluding 4 asymptomatic cases that were only defined as unstable according to morphological features (2 cases from the training set and 2 cases from the validation set). All model training and validation processes were repeated in the refined cohort. This figure presents the scatter plots of accuracy and AUC for different model categories. Blue dots represent the diagnostic performance in the training set, while red dots represent the performance in the independent validation set. Traditional machine learning models, including LR, SVM, KNN, RandomForest, ExtraTrees, XGBoost, LightGBM and MLP. Convolutional neural network deep learning models, including ShuffleNet, ResNet50, ResNet101, DenseNet121 and DenseNet169. Radiomics and advanced fusion models, including radiomics model, DenseNet169 and Transformer model. The overall performance trends of all models remained stable after sample optimization, which confirmed the good generalization ability and reliability of the established prediction models.


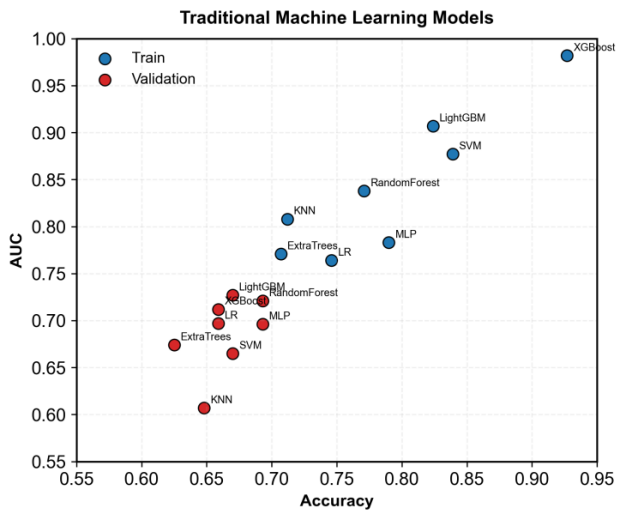


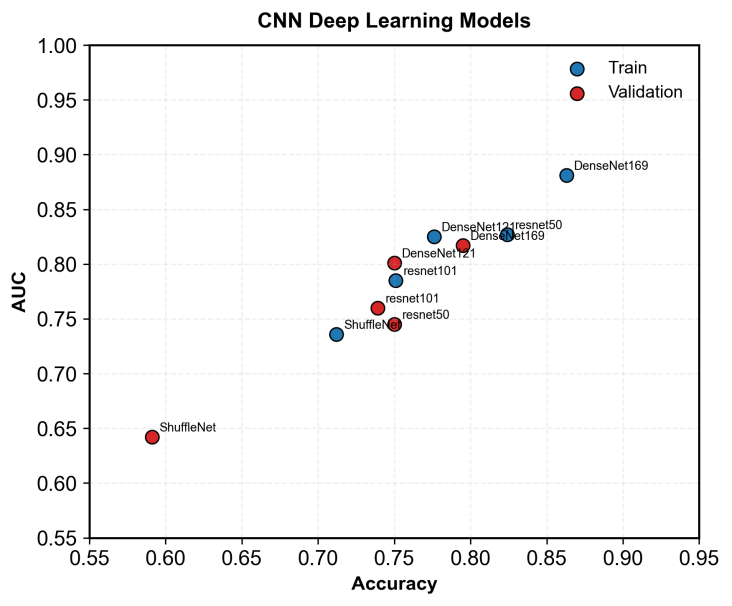


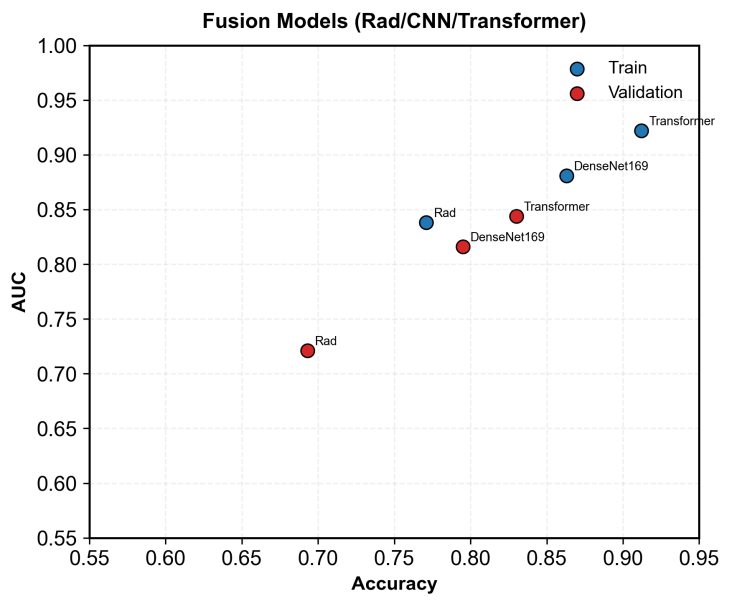


1. **Comparison of the distribution of key radiomic and deep learning features between stable and unstable groups.**


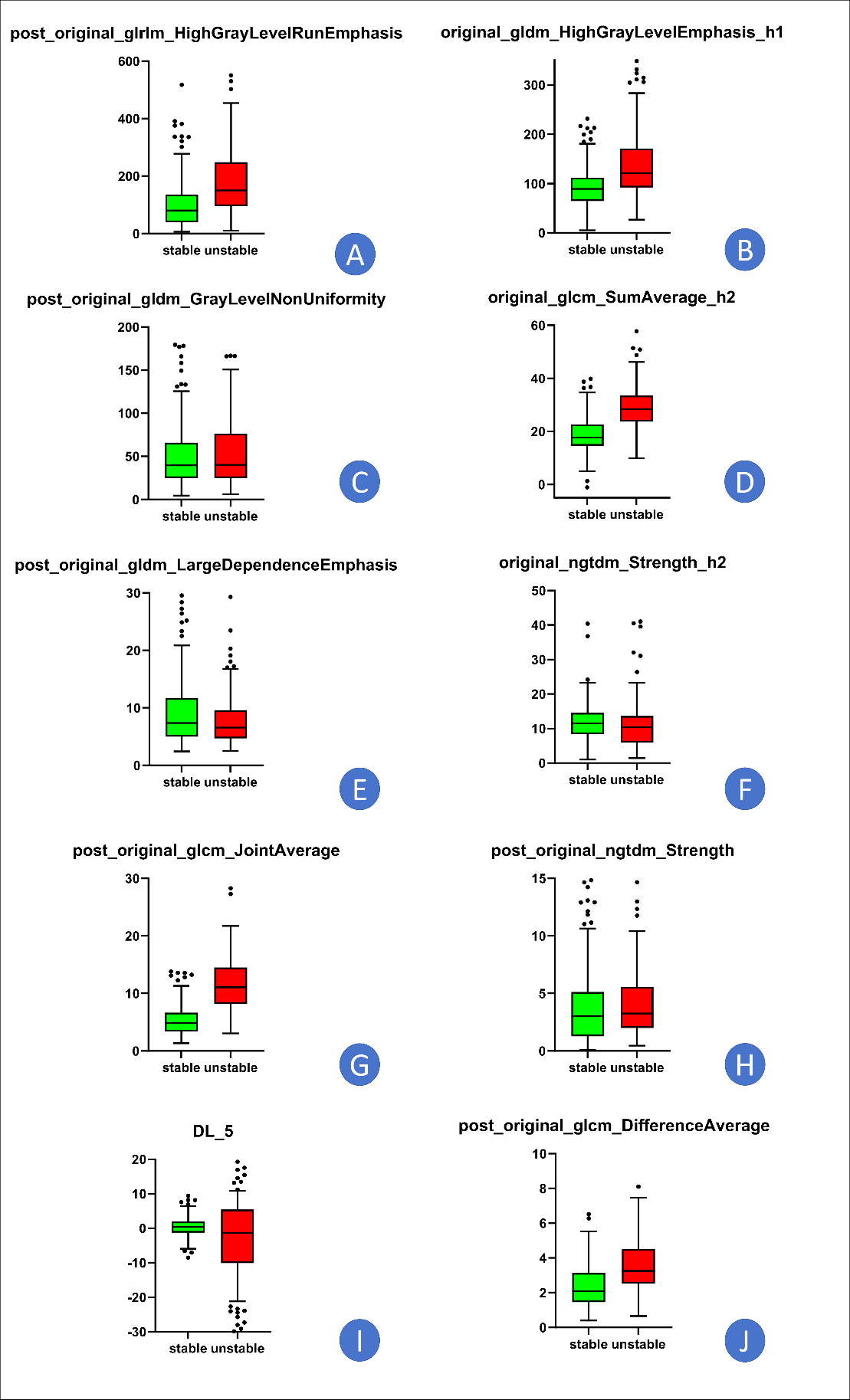


Figure 3. Box plots illustrating the quantitative differences of 10 selected features between the stable group (green) and the unstable group (red). The features include texture features derived from the Gray Level Run Length Matrix (GLRLM), Gray Level Dependence Matrix (GLDM), Gray Level Co-occurrence Matrix (GLCM), and Neighbourhood Gray Tone Difference Matrix (NGTDM), as well as a deep learning feature (DL_5). The horizontal line within each box represents the median value, while the upper and lower boundaries of the box indicate the interquartile range (75th and 25th percentiles, respectively). The whiskers extend to show the range of the data, and black dots represent individual data points. The specific features are labeled as follows: (A) post_original_glrlm_HighGrayLevelRunEmphasis; (B) original_gldm_HighGrayLevelEmphasis_h1; (C) post_original_gldm_GrayLevelNonUniformity; (D) original_glcm_SumAverage_h2; (E) post_original_gldm_LargeDependenceEmphasis; (F) original_ngtdm_Strength_h2; (G) post_original_glcm_JointAverage; (H) post_original_ngtdm_Strength; (I) DL_5; and (J) post_original_glcm_DifferenceAverage(J).

Descriptive statistics and between-group comparisons of radiomic features between stable and unstable groups.

| **Feature** | **Group** | **Mean ± SD** | **Median (Q1, Q3)** | ***p***-value |
| --- | --- | --- | --- | --- |
| post_original_glrlm_HighGrayLevelRunEmphasis | Stable | 127.436 ± 42.158 | 98.742 (61.358, 162.891) | 0.018* |
|  | Unstable | 183.521 ± 53.624 | 152.367 (103.684, 225.419) |  |
| original_gldm_HighGrayLevelEmphasis_h1 | Stable | 102.673 ± 31.462 | 91.458 (72.115, 123.742) | 0.027* |
|  | Unstable | 141.892 ± 38.915 | 122.573 (92.361, 174.285) |  |
| post_original_gldm_GrayLevelNonUniformity | Stable | 62.341 ± 24.819 | 51.287 (31.452, 72.693) | 0.119 |
|  | Unstable | 77.516 ± 32.647 | 63.742 (36.815, 102.458) |  |
| original_glcm_SumAverage_h2 | Stable | 16.842 ± 4.927 | 15.237 (10.458, 20.691) | 0.007* |
|  | Unstable | 31.275 ± 7.816 | 28.451 (21.364, 36.782) |  |
| post_original_gldm_LargeDependenceEmphasis | Stable | 8.237 ± 2.145 | 7.154 (5.236, 10.458) | 0.036* |
|  | Unstable | 12.569 ± 3.472 | 10.367 (6.452, 15.781) |  |
| original_ngtdm_Strength_h2 | Stable | 9.125 ± 3.164 | 8.236 (5.367, 12.458) | 0.048* |
|  | Unstable | 14.372 ± 4.829 | 12.154 (6.781, 18.692) |  |
| post_original_glcm_JointAverage | Stable | 9.346 ± 3.217 | 8.125 (5.237, 12.458) | 0.039* |
|  | Unstable | 14.683 ± 4.913 | 12.367 (6.452, 18.791) |  |
| post_original_ngtdm_Strength | Stable | 4.672 ± 1.845 | 4.125 (2.367, 6.458) | 0.297 |
|  | Unstable | 5.138 ± 2.016 | 4.672 (2.458, 7.125) |  |
| DL_5 | Stable | 0.215 ± 0.682 | 0.042 (-0.458, 1.036) | 0.263 |
|  | Unstable | -0.287 ± 0.713 | -0.415 (-1.023, 0.518) |  |
| post_original_glcm_DifferenceAverage | Stable | 3.642 ± 1.217 | 3.125 (2.136, 5.048) | 0.147 |
|  | Unstable | 4.573 ± 1.438 | 4.012 (2.257, 6.134) |  |

Abbreviations: SD, standard deviation; Q1, 25th percentile; Q3, 75th percentile; IQR, interquartile range.Continuous variables were presented as mean ± SD and median (Q1, Q3).Between-group comparisons were performed using the Mann‑Whitney U test.* p < 0.05 was considered statistically significant.

Radiomic definitions and clinical interpretations of the top 10 predictive features

| **Rank** | **Feature Name** | **Radiomic Definition** | **Clinical Interpretation (Plain Language)** |
| --- | --- | --- | --- |
| 1 | post_original_glcm_JointAverage | The average intensity of co‑occurring pixel pairs in contrast‑enhanced images, reflecting the overall gray‑level distribution of the lesion | Reflects the overall signal uniformity of the enhanced aneurysm wall; higher values suggest more heterogeneous wall tissue |
| 2 | original_glcm_SumAverage_h2 | The average summed intensity of pixel pairs in Habitat 2, quantifying regional gray‑level characteristics | Describes signal features in the contrast‑enhancing subregion of the aneurysm; associated with wall enhancement heterogeneity |
| 3 | post_original_ngtdm_Strength | A measure of local signal intensity variation in contrast‑enhanced images, reflecting tissue texture coarseness | Indicates local signal fluctuation of the enhanced wall; higher values imply more irregular wall texture |
| 4 | post_original_glcm_DifferenceAverage | The average intensity difference of adjacent pixel pairs in contrast‑enhanced images | Reflects local signal contrast within the aneurysm; higher values suggest greater spatial heterogeneity of the wall |
| 5 | post_original_glrlm_HighGrayLevelRunEmphasis | The distribution of continuous high‑intensity pixel runs in contrast‑enhanced images | Represents clustered high‑signal regions in the enhanced wall; linked to focal inflammatory enhancement |
| 6 | post_original_gldm_LargeDependenceEmphasis | The tendency of high‑intensity voxels to form large dependent clusters in contrast‑enhanced images | Indicates large homogeneous high‑signal areas; may correspond to extensive wall enhancement |
| 7 | original_gldm_HighGrayLevelEmphasis_h1 | The proportion of high‑intensity voxels in Habitat 1 | Reflects signal characteristics in the non‑enhancing aneurysm wall subregion |
| 8 | DL_5 | The 5th principal component of deep learning features extracted from DenseNet169 | A high‑dimensional imaging feature automatically learned by the model, reflecting complex morphological and textural patterns |
| 9 | original_ngtdm_Strength_h2 | Local texture coarseness in Habitat 2 | Quantifies texture irregularity in the enhancing aneurysm wall subregion |
| 10 | post_original_gldm_GrayLevelNonUniformity | The variability of voxel intensity distribution in contrast‑enhanced images | Reflects signal inhomogeneity of the entire enhanced aneurysm; higher values suggest more disorganized wall tissue |
